# Strain-specific joint invasion and colonization by Lyme disease spirochetes is promoted by outer surface protein C

**DOI:** 10.1371/journal.ppat.1008516

**Published:** 2020-05-15

**Authors:** Yi-Pin Lin, Xi Tan, Jennifer A. Caine, Mildred Castellanos, George Chaconas, Jenifer Coburn, John M. Leong

**Affiliations:** 1 Department of Molecular Biology and Microbiology, Tufts University School of Medicine, Boston, Massachusetts, United States of America; 2 Division of Infectious Diseases, New York State Department of Health, Wadsworth Center, Albany, New York, United States of America; 3 Departments of Biochemistry & Molecular Biology and Microbiology, Immunology & Infectious Diseases, Snyder Institute for Chronic Diseases, University of Calgary, Calgary, Alberta, Canada; 4 Division of Infectious Diseases, and Center for Infectious Disease Research, Medical College of Wisconsin, Milwaukee, Wisconsin, United States of America; University of Montana, UNITED STATES

## Abstract

Lyme disease, caused by *Borrelia burgdorferi*, *B*. *afzelii* and *B*. *garinii*, is a chronic, multi-systemic infection and the spectrum of tissues affected can vary with the Lyme disease strain. For example, whereas *B*. *garinii* infection is associated with neurologic manifestations, *B*. *burgdorferi* infection is associated with arthritis. The basis for tissue tropism is poorly understood, but has been long hypothesized to involve strain-specific interactions with host components in the target tissue. OspC (outer surface protein C) is a highly variable outer surface protein required for infectivity, and sequence differences in OspC are associated with variation in tissue invasiveness, but whether OspC directly influences tropism is unknown. We found that OspC binds to the extracellular matrix (ECM) components fibronectin and/or dermatan sulfate in an OspC variant-dependent manner. Murine infection by isogenic *B*. *burgdorferi* strains differing only in their *ospC* coding region revealed that two OspC variants capable of binding dermatan sulfate promoted colonization of all tissues tested, including joints. However, an isogenic strain producing OspC from *B*. *garinii* strain PBr, which binds fibronectin but not dermatan sulfate, colonized the skin, heart and bladder, but not joints. Moreover, a strain producing an OspC altered to recognize neither fibronectin nor dermatan sulfate displayed dramatically reduced levels of tissue colonization that were indistinguishable from a strain entirely deficient in OspC. Finally, intravital microscopy revealed that this OspC mutant, in contrast to a strain producing wild type OspC, was defective in promoting joint invasion by *B*. *burgdorferi* in living mice. We conclude that OspC functions as an ECM-binding adhesin that is required for joint invasion, and that variation in OspC sequence contributes to strain-specific differences in tissue tropism displayed among Lyme disease spirochetes.

## Author summary

Infection by different Lyme disease bacteria is associated with different manifestations, such as cardiac, neurologic, or, in the case of *B*. *burgdorferi*, the major cause of Lyme disease in the U.S., joint disease. The basis for these differences is unknown, but likely involve strain-specific interactions with host components in the target tissue. The sequence of the outer surface lipoprotein OspC varies with the strains, and we found that this variation influences the spectrum of host extracellular matrix components recognized. Infection of mice with strains that are identical except for *ospC* revealed that OspC variants that differ in binding spectrum promote infection of different tissues. A strain producing OspC invaded and colonized the joint in living animals, but an altered OspC protein incapable of binding tissue components did not. Thus, tissue-binding by OspC is critical for infection and joint invasion, and OspC variation directly influences tissue tropism.

## Introduction

Lyme disease, an infection found throughout the Northern Hemisphere and caused by spirochetes of the genus *Borrelia* (recently renamed *Borreliella* [1]) that are transmitted by *Ixodes* ticks, affects greater than 30,000 people each year in the U.S. alone [2]. An initial skin infection develops at the site of the tick bite, often giving rise to the characteristic ‘bull’s eye’ rash, erythema migrans. Some Lyme disease strains appear to cause only local infections, but many strains spread to other tissues, such as the heart, joints, nervous system or distant skin sites, in the subsequent days or weeks. Weeks to months after initial infection, chronic or late Lyme disease may display distinct clinical syndromes. In the U.S., Lyme borreliosis is caused primarily by *B*. *burgdorferi* and commonly features arthritis [3]. In contrast, in Europe and Asia, Lyme disease can also be caused by *B*. *afzelii* and *B*. *garinii*, which commonly give rise to late stage infections featuring dermatologic or neurologic manifestations, respectively [4–7].

Attachment to host tissues is viewed as an essential early step in colonization [8, 9]. Lyme disease *Borrelia spp*. are highly adhesive and produce at least 19 outer surface proteins that recognize host cells or extracellular matrix (ECM) [9–12]. Remarkably, given the number of these adhesins, mutants that lack one are often detectably diminished both for cell or ECM attachment *in vitro* and for infectivity during experimental animal infection [13–16]. In several instances, adhesin-deficient mutants are selectively defective for colonization of specific tissues. For example, a *B*. *burgdorferi* mutant deficient for production of the fibronectin-binding adhesin RevA [17] is diminished for colonization of the heart [18, 19], and a mutant deficient in glycosaminoglycan- (GAG-) binding by the surface lipoprotein BBK32 is diminished for colonization of the joint [20].

These findings notwithstanding, the basis for the differences in invasiveness and tissue tropism among Lyme disease spirochetes is poorly understood. Lyme disease spirochetes encode several adhesins that are variable in sequence among different strains, raising the possibility that variable adhesive surface proteins contribute to observed strain- or species-specific differences in tissue tropism and clinical manifestation. In fact, variants of DbpA, an adhesin that binds to the proteoglycan decorin and the glycosaminoglycan (GAG) dermatan sulfate, exhibit differences in GAG- and decorin-binding *in vitro*, and isogenic *B*. *burgdorferi* strains that differed only in the coding sequence of *dbpA* display differences in tissue tropism upon infection of laboratory mice [21–24].

OspC is a 22 kDa lipoprotein on the surface of *B*. *burgdorferi* that is not detected when the spirochete is in non-feeding ticks but produced upon the initiation of feeding [25]. OspC plays an essential role in the first days of mammalian infection [26–28]. Furthermore, other than VlsE, which undergoes extensive antigenic variation during mammalian infection, OspC displays the highest inter-strain variability of any Lyme disease spirochete proteins [29–31]. The central portion of OspC is highly variable [32, 33] and OspC variants fall into one of 22 classes, termed A through U, with at least 8% variability separating different groups [34–36]. Notably, a subset of OspC classes are produced by *Borrelia* strains and species associated with disseminated infections in humans and/or mice [7, 34, 35, 37–40]. OspC binds to the host protease precursor, plasminogen, *in vitro* [37, 41] and inhibits the phagocytosis of spirochetes by macrophages *ex vivo* [42], although direct evidence linking these activities of OspC to infectivity in the mammalian host remains to be developed. Recently, OspC of *B*. *burgdorferi* strain B31 was shown to promote bloodstream survival in mice and inhibit activation of the classical and lectin complement pathways *in vitro* [43]. The formation of the C4b2a complex by factors C4 and C2 is critical for the activation of these pathways, and this OspC variant and C2 were shown to bind to human C4b in a mutually exclusive manner. However, an OspC variant that does not bind human C4b still promotes bloodstream survival in mice [43], indicating that the role that OspC plays during mammalian infection remains incompletely understood.

OspC is orthologous to Vsps (variable small proteins), which are antigenically variable surface proteins encoded by relapsing fever borreliae, such as *Borrelia turicatae* and *B*. *hermsii* [44, 45], and shares overlapping function with a member of this family, Vtp, that is expressed upon acquisition of the blood meal [46]. *B*. *turicatae* variant Vsp2 but not Vsp1 recognizes GAGs [47], and *B*. *turicatae* production of Vsp2 rather than Vsp1 is associated with seven- to eight-fold better colonization of the blood and joint [48]. These data suggest that Vsps, and by inference, perhaps OspC, influence tissue tropism of pathogenic *Borrelia* by binding ECM components such as GAGs. Finally, when a library of phages displaying *B*. *burgdorferi* protein fragments on the surface was inoculated intravenously (*i*.*v*.) into mice, phages producing OspC peptides were enriched in the heart and joints [49], reinforcing the possibility that OspC may function as an adhesin. However, evidence that OspC promotes spirochetal interaction with host cells or ECM is lacking. Furthermore, colonization of target tissue is a multistep process [8, 9, 50], and the pathogenic consequence of a putative interaction between surface-localized OspC and host tissue is completely unexplored.

In the current study, we found that OspC confers variant-specific binding of the bacteria to dermatan sulfate and/or fibronectin. Murine infection by isogenic *B*. *burgdorferi* strains that differ only in *ospC* coding sequence revealed that OspC variation results in differences in joint colonization. An OspC mutant incapable of binding either dermatan sulfate or fibronectin was dramatically deficient in tissue colonization and found in tissues at levels no higher than an OspC-defective strain. Intravital microscopy revealed that this OspC mutant, in contrast to wild type OspC, was defective in promoting vascular transmigration into knee joint tissues by *B*. *burgdorferi* in living animals. We conclude that OspC functions as an adhesin that promotes joint invasion, and that the variation in OspC sequence contributes to strain-specific differences in tissue tropism displayed by Lyme disease spirochetes.

## Results

### Recombinant OspC protein of *B*. *burgdorferi* sensu stricto N40-D10/E9 binds to fibronectin and dermatan sulfate

**Fig 1:**
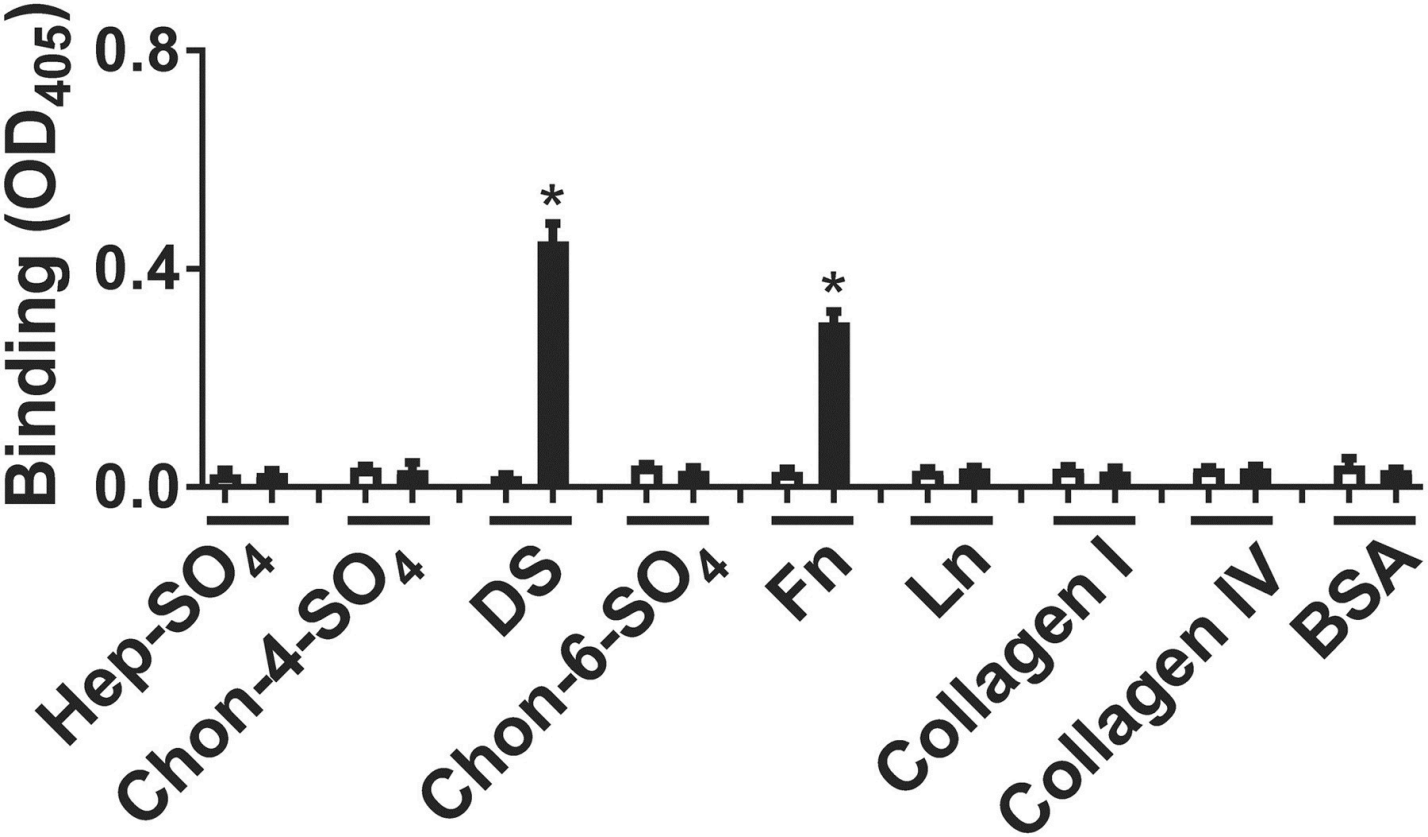
Recombinant OspC_N40-D10/E9_ binds to fibronectin and dermatan sulfate. Quadruplicate wells were incubated with 10 μg/ml of heparan sulfate (Hep-SO_4_), chondroitin-4-sulfate (Chon-4-SO_4_), dermatan sulfate (DS), chondroitin-6-sulfate (Chon-6-SO_4_), fibronectin (Fn), laminin (Ln), type I collagen (Collagen I), type IV collagen (Collagen IV) or BSA (see Materials and Methods). Two μM recombinant GST-tagged OspC_N40-D10/E9_ (black bars) or GST (negative control; white bars) was then added to the wells. Bound protein was measured by ELISA and mean OD_405_ ± SEM was determined. Asterisks indicate that binding of GST-OspC to dermatan sulfate or fibronectin was significantly (*p* ≤ 0.05) greater than binding by GST by Student’s t-test. Shown is a representative of three independently performed experiments.

Previous work has revealed that many Lyme borreliae surface proteins bind ECM components [17, 50–57], and OspC was previously identified as a candidate adhesin [49], so we generated a GST fusion protein of OspC that encompasses the mature sequence but lacks the (cleaved) signal peptide of *B*. *burgdorferi* strain N40-D10/E9 (“OspC_N40-D10/E9_“) and assessed its ability to bind a number of ECM components, including fibronectin, laminin, collagen, and a variety of GAGs immobilized in microtiter wells. ELISA detection of bound GST-OspC_N40-D10/E9_ using anti-GST antibody revealed that fibronectin and dermatan sulfate GAG were recognized by GST-OspC_N40-D10/E9_ but not GST (Fig 1). No other substrates tested were recognized by this protein.

**Fig 2:**
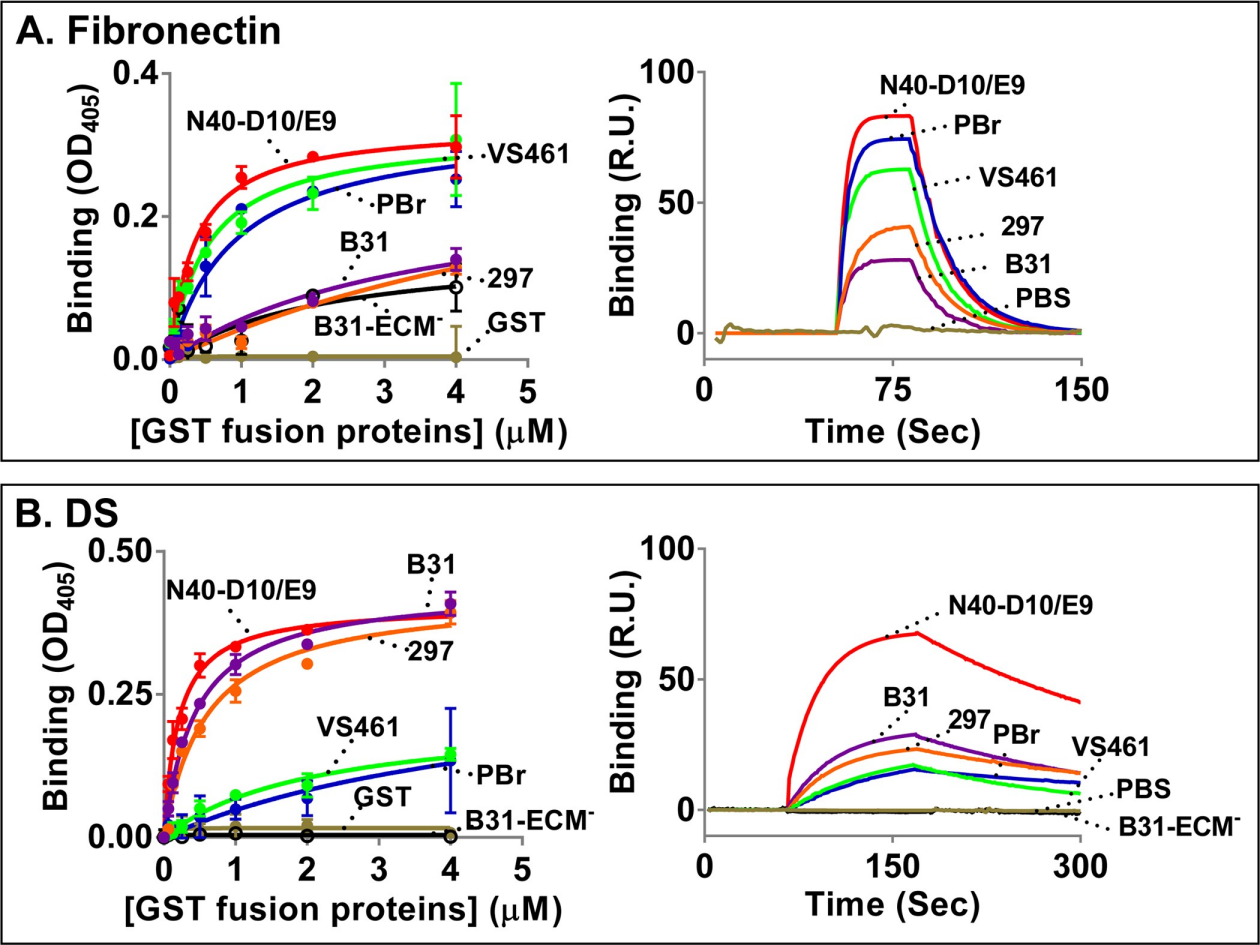
Recombinant OspC variants exhibit distinct fibronectin- and dermatan sulfate-binding activities. **(A) Left panel:** Quadruplicate wells were incubated with 10 μg/ml of fibronectin. The indicated concentrations of various recombinant GST-tagged OspC variants, including OspC_B31_ (“B31”), OspC_N40-D10/E9_ (“N40-D10/E9”), OspC_297_ (“297”), OspC_PBr_ (“PBr”), OspC_VS461_ (“VS461”), OspC_B31-ECM_^-^ (“B31-ECM^-^”), or GST (as a negative control), were then added to the wells. The protein binding was quantified by ELISA. The *K*_D_ values were obtained from the average of three independent experiments and shown on Table 1. Shown is a representative of three experiments with numbers indicating the mean ± standard deviation derived from quadruplicate wells. **Right panel:** Ten μg of fibronectin in 10 μl was allowed to flow through CM5 chips. 15.625 to 500 nM of the indicated untagged OspC protein was then flowed over the chips. Binding was measured in response units (R.U.) by SPR. Shown is the binding of 500nM of indicated OspC proteins to fibronectin from one experiment as a representative of six experiments performed on three different occasions. The *K*_D_ values were obtained from average of the six experiments and shown in Table 1. **(B) Left panel:** Quadruplicate wells were incubated with 10 μg/ml of dermatan sulfate (DS). Indicated GST-tagged OspC variants were then added to the wells, as described in Panel A. The *K*_D_ values were obtained from the average of three independent experiments and shown in Table 1. Shown is a representative of the three experiments with numbers indicating the mean ± standard deviation derived from quadruplicate wells. **Right panel:** Ten μg of biotinylated dermatan sulfate in 10 μl was allowed to flow through SA chips.15.625 to 500 nM of the indicated untagged OspC variants was then flowed over the chips, as described in panel A. Shown is the binding of indicated OspC proteins to dermatan sulfate from one experiment as a representative of six experiments performed on three different occasions. The *K*_D_ values were obtained from average of the six experiments and shown in Table 1.

To estimate the dissociation constants for binding of dermatan sulfate or fibronectin to GST-OspC_N40-D10/E9_, varying concentrations of GST-OspC_N40-D10/E9_ or, as a control, GST alone, were incubated in wells coated with purified dermatan sulfate or fibronectin, and binding was determined by ELISA (Fig 2, left panels). OspC_N40-D10/E9_ bound to dermatan sulfate with an estimated *K*_D_ of 0.16μM and to fibronectin with an estimated *K*_D_ of 0.35μM (Fig 2, left panels, and Table 1). To independently assess the dissociation constants of OspC_N40-D10/E9_, binding to dermatan sulfate or fibronectin was tested by surface plasmon resonance (SPR; Fig 2, right panels). These experiments revealed *k*_on_ and *k*_off_ rates that result in calculated *K*_D_‘s of binding of OspC_N40-D10/E9_ to dermatan sulfate or fibronectin to be 0.25μM or 0.30μM, respectively, i.e. consistent with the *K*_D_’s determined by ELISA (Table 1).

**Table 1:** OspC variants differ in binding to dermatan sulfate and fibronectin.

		ELISA	----- Surface Plasmon Resonance -----		
OspC variant	Ligand	K_D_ (μM)	K_D_ (μM)	k_on_ (10^4^s^-1^M^-1^)	k_off_ (s^-1^)
***B*. *burgdorferi***					
**OspC_N40-D10/E9_**	**Derm S0_4_**	**0.16±0.05**	**0.27±0.07**^a^	4.64±1.94^a^	0.01±0.003^a^
	**Fibronectin**	**0.35±0.03**	**0.30±0.07**^a^	45.08±14.94^a^	0.13±0.04^a^
**OspC_B31_**	**Derm S0_4_**	**0.40±0.07**	**0.57±0.17**^a^	1.71±0.17^a^	0.01±0.004^a^
	**Fibronectin**	**3.46±1.46**^b^	**9.21±4.04**^a^^,^^b^	1.99±0.70^a^	0.13±0.05^a^
**OspC_B31-ECM-_**	**Derm S0_4_**	**n.b.**^c^	**n.b.**^c^	n.b.^c^	n.b.^c^
	**Fibronectin**	**2.14±1.82**^b^	**n.d.**^d^	n.d.^d^	n.d.^d^
**OspC_297_**	**Derm S0_4_**	**0.60±0.13**	**0.75±0.32**^a^	1.69±0.35^a^	0.01±0.002^a^
	**Fibronectin**	**2.78±1.46**^b^	**1.75±0.40**^a^^,^^b^	3.68±0.24^a^	0.06±0.005^a^
***B*. *garinii***					
**OspC_PBr_**	**Derm S0_4_**	**5.53±3.92**^b^	**9.97±2.19**^a^^,^^b^	0.040±0.001^a^	0.003±0.001^a^
	**Fibronectin**	**0.90±0.20**	**0.71±0.08**^a^	19.20±5.95^a^	0.13±0.04^a^
***B*. *afzelii***					
**OspC_VS461_**	**Derm S0_4_**	**2.01±0.31**^b^	**4.21±1.10**^a^^,^^b^	0.042±0.002^a^	0.002±0.001^a^
	**Fibronectin**	**0.52±0.08**	**0.80±0.34**^a^	15.06±3.94^a^	0.09±0.03^a^

All values represent the mean ± SEM of three experiments.

^a^The *K*_*D*_ values were obtained from the average k_off_ divided k_on_ from each run.

^b^The values should be considered estimates due to the weakness of the interaction.

^c^No binding activity was detected.

^d^Not determined.

### Recombinant OspC variants exhibit distinct fibronectin- and dermatan sulfate-binding activities

OspC demonstrates sequence variability among different strains [32]. To determine if OspC variants among strains of Lyme disease spirochetes differ in their adhesive activity to dermatan sulfate and fibronectin, GST-OspC proteins from OspC of *B*. *burgdorferi* strains B31-A3 (OspC_B31_) and 297 (OspC_297_), *B*. *garinii* strain PBr (OspC_PBr_), and *B*. *afzelii* strain VS461 (OspC_VS461_), all of which belong to different OspC classes [7, 36] were tested for binding to dermatan sulfate and fibronectin by ELISA whereas these OspC variants without GST tags were examined for the binding to these ECM ligands by SPR (Fig 2). Estimated dissociation constants were calculated for each interaction using both ELISA and SPR, and the two methods gave highly consistent results (Table 1). Interestingly, the OspC variants demonstrated considerable differences in their recognition of fibronectin and dermatan sulfate: OspC_VS461_ and OspC_PBr_ bound preferentially to fibronectin (*K*_D_~0.5–0.9 μM) as compared to dermatan sulfate (*K*_D_ ~2.0–10.0 μM), whereas OspC_B31_ and OspC_297_ bound preferentially to dermatan sulfate (*K*_D_ ~0.4–0.8 μM) as compared to fibronectin (*K*_D_ ~1.7–9.2 μM) (Fig 2 and Table 1).

### *B*. *burgdorferi* OspC promotes adhesion to the joint vasculature in a one-hour intravenous inoculation model

**Fig 3:**
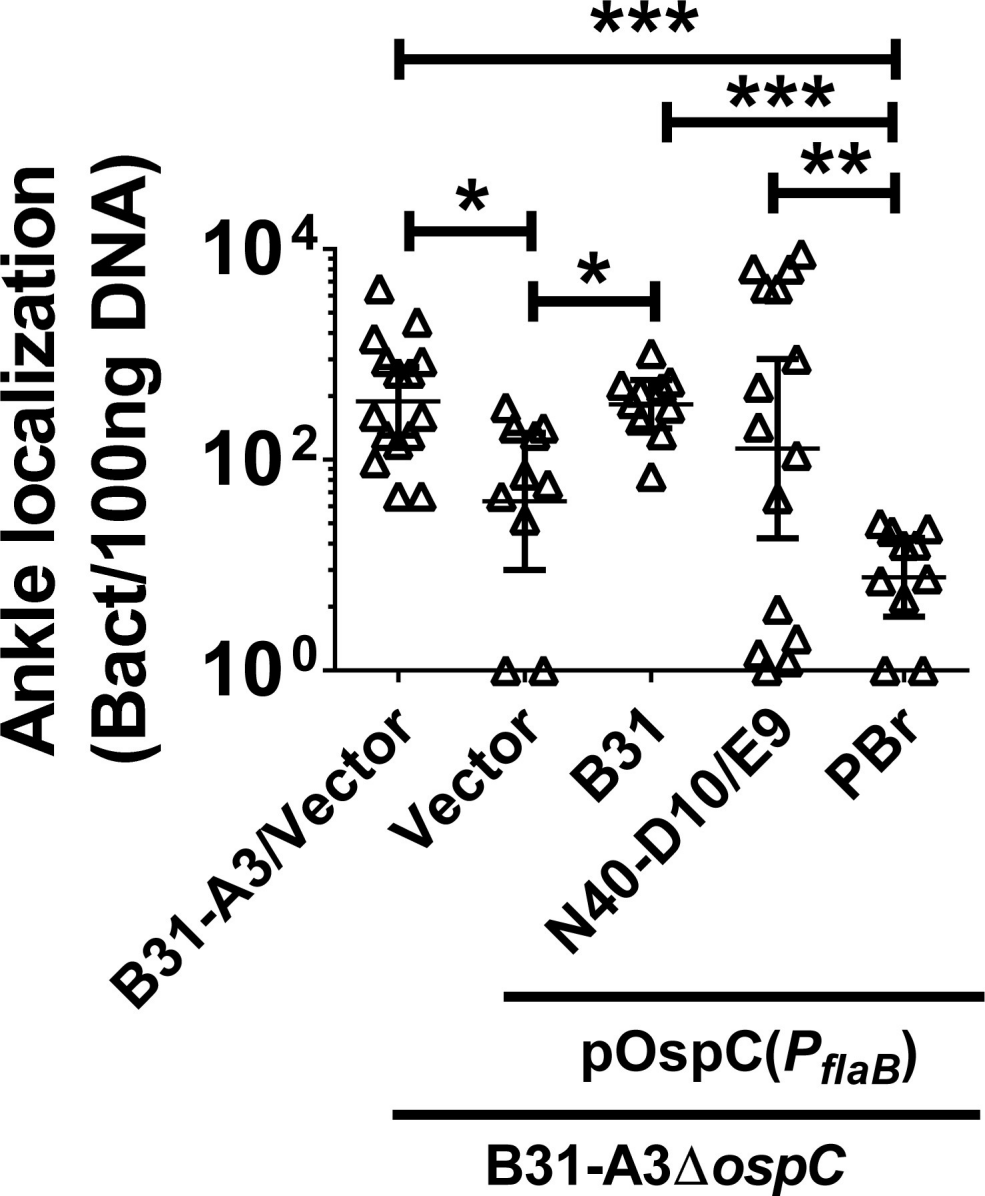
OspC promotes ankle joint localization of spirochetes in an allele-specific manner one hour after intravenous inoculation. C3H/HeN mice were inoculated retro-orbitally with infectious *B*. *burgdorferi* strain B31-A3 with the vector pBSV2G (“B31-A3/Vector”), B31-A3*ΔospC* with the vector (“Vector”), or B31-A3*ΔospC* producing OspC from B31-A3 (“B31”), *B*. *burgdorferi* strain N40 clone D10/E9 (“N40-D10/E9”), or *B*. *garinii* strain PBr (“PBr”). After 1 hr, perfused tissues were collected and bacterial burdens were quantified by qPCR. Data are shown as the geometric mean ± 95% confidence interval. Statistical significance was determined using ANOVA with the Kruskal-Wallis test followed by the two-stage step-up method of Benjamini, Krieger and Yekutieli. * = P < 0.05, ** = P < 0.01, *** = P < 0.001. N = 10 or 15 mice per *B*. *burgdorferi* strain.

Because OspC was selected for binding to the vasculature of living mice shortly after *i*.*v*. inoculation of a phage display library [49], we tested the role of the protein as produced by *B*. *burgdorferi* in mediating very early interactions with the vasculature of the ankle joint in our 1 hr *i*.*v*. inoculation model [18]. We produced OspC variants in an *ospC*-deficient mutant of *B*. *burgdorferi* strain B31-A3 [28], utilizing pBSV2G-derived *ospC*-complementing plasmids that are identical except for their *ospC* coding sequences (i.e. *ospC*_*B31*_, *ospC*_*N40-D10/E9*_, or *ospC*_*PBr*_). For this short-term infection model, all *ospC* genes were cloned 3’ to the *flaB* promoter to ensure protein production by *in vitro* cultivated bacteria. One hour after inoculation, all of the strains were able to localize to the joint at detectable levels, consistent with the expression of lipoproteins such as BBK32 and DbpA, which promote joint adhesion [9, 20, 22, 50]. Nevertheless, OspC_B31_ and OspC_N40-D10/E9_ promoted *B*. *burgdorferi* adhesion to the vasculature of the ankles in living mice at levels similar to the parental B31-A3, while the numbers of bacteria producing no OspC (*ΔospC*) or OspC_PBr_ were significantly reduced in comparison (Fig 3).

### Generation of an OspC mutant that is defective for binding to dermatan sulfate and fibronectin

To better study the contribution of ECM binding by OspC, targeted mutations were made to generate recombinant OspC protein lacking both fibronectin- and dermatan sulfate-binding activity. We focused on a 61-residue segment in the central region of OspC that was previously found to be common to all surface display phage enriched for tissue targeting after *i*.*v*. infection of mice (S1 Fig) [49]. Basic residues have been shown to be critical for activity by other GAG-binding proteins [58], and of five residues 116, 121, 123, 128, and 129, located in α-helix 3 and loop 4, all are lysines in the dermatan sulfate-binding variants OspC_B31_ and OspC_297_, and four of the five are lysines in OspC_N40-D10/E9_, which also binds dermatan sulfate (S2A and s2B Fig). In contrast, only three and two of these residues are lysines in the variants OspC_VS461_ and OspC_PBr_, respectively, which do not efficiently bind to dermatan sulfate. Wild type OspC_B31_ binds inefficiently to fibronectin (*K*_D_ ~3.5 μM, Table 1), so to generate an OspC derivative lacking both high-level fibronectin- and dermatan sulfate-binding activity, the five OspC_B31_ lysine residues in this region of OspC_B31_ were mutated to methionine. Maintenance of secondary structure of this derivative, which we termed “OspC_B31-ECM_^-^”, was confirmed by circular dichroism (CD) analysis (S2C Fig). In addition, OspC_B31-ECM_^-^ and wild type OspC_B31_ bound to C4b indistinguishably, indicating that the structure of the protein is not dramatically altered by the substitutions and that the lysine residues are not required for C4b-binding activity (S2D Fig). ELISA analysis demonstrated that the fibronectin-binding activity of the mutant protein remained poor (*K*_D_ ~2.1 μM), and its dermatan sulfate-binding activity was undetectable (Fig 2 and Table 1).

### *B*. *burgdorferi* producing OspC variants differ in their ability to bind to fibronectin and dermatan sulfate

We investigated whether the different degrees of dermatan sulfate- or fibronectin-binding by OspC variants in purified recombinant form correspond to their ability to mediate spirochetal binding to these substrates when expressed on the surface of the bacterium. To interrogate a set of OspC variants that together encompass a wide variety of binding phenotypes, we analyzed: (a) OspC_B31-ECM_^-^, which recognizes neither fibronectin or dermatan sulfate efficiently; (b) OspC_B31_, which efficiently recognizes dermatan sulfate but not fibronectin; (c) OspC_N40-D10/E9_, which efficiently binds both fibronectin and dermatan sulfate; and (d) OspC_PBr_, which recognizes fibronectin but not dermatan sulfate (Table 1). We produced these OspC variants in the *ospC*-deficient mutant of *B*. *burgdorferi* strain B31-A3 [28], utilizing pBSV2G-derived *ospC*-complementing plasmids that are expressed by the *ospC*_*B31*_ promoter in order to mimic the expression level of endogenous *ospC*, but differ in their *ospC* coding sequences. (These plasmids encoding *ospC*_*B31*_, *ospC*_*B31-ECM*_^-^, *ospC*_*N40-D10/E9*_, and *ospC*_*PBr*_ were termed pOspC_B31_, pOspC_B31-ECM_^-^, pOspC_N40-D10/E9_, and pOspC_PBr_, respectively). Flow cytometric quantitation of the surface production levels of all OspC variants or mutants (see Materials and Methods) indicated that all were efficiently displayed on the spirochetal surface, indistinguishably from the level of OspC on the surface of *B*. *burgdorferi* strain B31-A3 carrying pBSV2G (vector) alone (S3 Fig).

**Fig 4:**
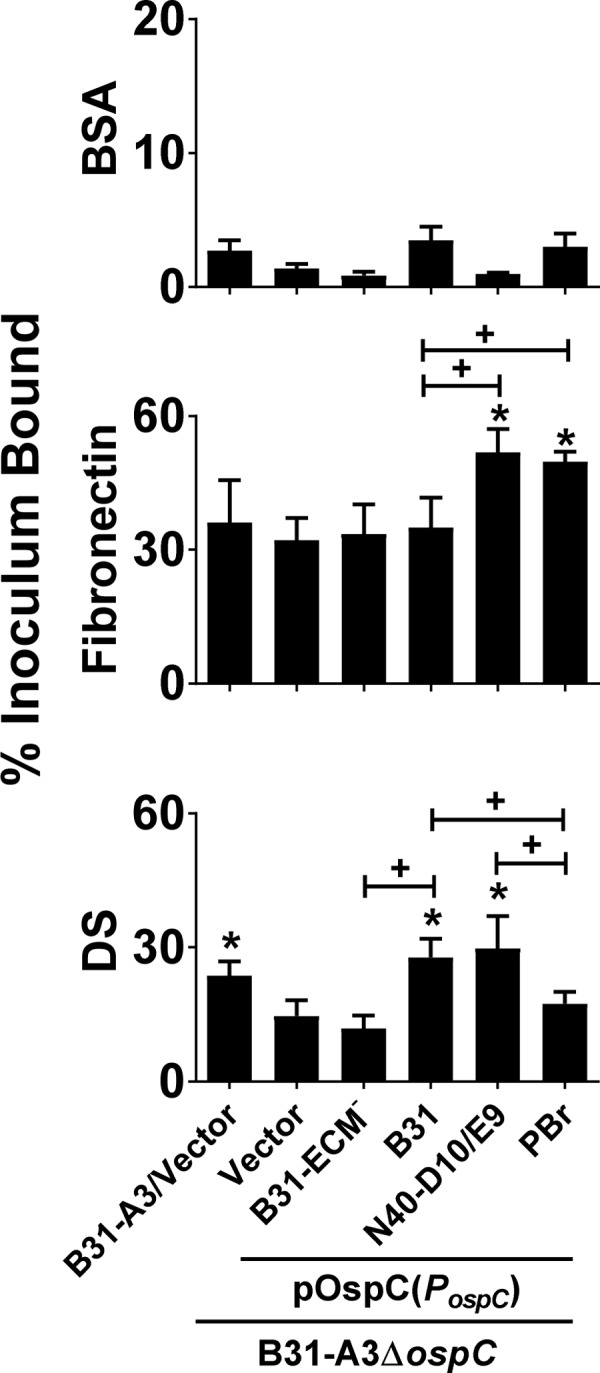
*B*. *burgdorferi* producing OspC variants differ in their ability to bind to fibronectin and dermatan sulfate. Binding of radiolabeled *B*. *burgdorferi* B31-A3/pBSV2G (“B31-A3/Vector”), *ospC*-deficient strain B31-A3Δ*ospC*/pBSV2G (“Vector”), or the deletion strain bearing a plasmid encoding the indicated OspC variants to BSA **(Top panel)**, fibronectin **(Middle panel),** or dermatan sulfate (DS) **(Bottom panel)** was determined (see Materials and Methods). Each bar represents the mean of four independent determinations ± SEM. Statistical significance was determined using ANOVA with the Kruskal-Wallis test followed by the two-stage step-up method of Benjamini, Krieger and Yekutieli. Significant (*p* < 0.05) differences in binding relative to the OspC-deficient strain B31-A3Δ*ospC*/pBSV2G (‘‘*”) or between two strains relative to each other (‘‘+”) are indicated.

These strains, as well as *B*. *burgdorferi* strain B31-A3Δ*ospC*/pBSV2G, were radiolabeled and incubated in wells coated with purified fibronectin. After washing, stably bound bacteria were quantitated by scintillation counting. BSA-coated wells, used as a negative control, were found to consistently immobilize less than 5% of the inoculum (Fig 4, top panel). *B*. *burgdorferi* strain B31-A3/pBSV2G bound to fibronectin significantly better than to control wells (Fig 4, “B31-A3/Vector”, middle panel), consistent with the presence of outer surface proteins, such as BBK32, RevA, and BB0347, that promote binding to this ECM protein [17, 50–52]. As expected, given that GST-OspC_B31_ does not recognized fibronectin, strain B31-A3Δ*ospC*/pBSV2G (Fig 4, “Vector”, middle panel) demonstrated no defect in fibronectin binding compared to wild type strain B31-A3, and ectopic production of OspC_B31_ by this strain did not enhance fibronectin binding (Fig 4, “B31”, middle panel). Similarly, ectopic production of OspC_B31-ECM_^-^ by strain B31-A3Δ*ospC* did not enhance fibronectin binding (Fig 4, “B31-ECM^-^”, middle panel). In contrast, B31-A3Δ*ospC* harboring plasmids producing OspC_N40-D10/E9_ or OspC_PBr_, both of which as GST-fusion proteins efficiently recognize fibronectin, resulted in enhanced fibronectin-binding by B31-A3Δ*ospC* (Fig 4, “N40-D10/E9” and “PBr”, middle panel). Hence, the fibronectin-binding properties of the OspC variants determined using recombinant protein are reflected in their ability to promote fibronectin-binding by *B*. *burgdorferi*.

These strains were also tested for binding to immobilized dermatan sulfate. *B*. *burgdorferi* strain B31-A3/pBSV2G (Fig 4, “B31-A3/Vector”, bottom panel) bound to dermatan sulfate significantly better (23% vs. 14%) than did B31-A3Δ*ospC* harboring control vector (Fig 4, “Vector”, bottom panel), indicating that OspC produced by this strain promotes spirochetal binding to dermatan sulfate. Notably, the lower level of dermatan sulfate-binding by the *ospC*-deficient derivative of B31-A3 was still higher than binding to control wells, consistent with the presumed expression of other dermatan sulfate-binding proteins such as BBK32, Bgp, DbpB and DbpA [54, 55, 59–61]. Ectopic expression of OspC_B31_ from pOspC_B31_ complemented the relative defect in dermatan sulfate binding by the *ospC*-deficient strain (Fig 4, “B31“, bottom panel), and complementation of binding required dermatan sulfate-binding activity because ectopic production of OspC_B31-ECM_^-^, which does not exhibit dermatan sulfate-binding activity as a GST fusion protein, did not result in enhanced binding (Fig 4, “B31-ECM^-^“, bottom panel). Similarly, ectopic expression of OspC_PBr_, which also lacks dermatan-sulfate binding as a recombinant protein, did not increase binding by B31-A3Δ*ospC* (Fig 4, “PBr”, bottom panel). Finally, as predicted given the ability of GST-OspC_N40-D10/E9_ to bind dermatan sulfate, production of OspC_N40-D10/E9_ by B31-A3Δ*ospC* significantly enhanced GAG binding. Thus, the fibronectin- and dermatan sulfate-binding activities of each OspC variant that were determined by ELISA and SPR (Table 1) correlated with their ability to promote spirochetal binding to the two substrates.

### OspC of *B*. *burgdorferi* sensu stricto strains B31-A3 and N40-D10/E9, but not *B*. *garinii* strain PBr, promote joint colonization and swelling

**Fig 5:**
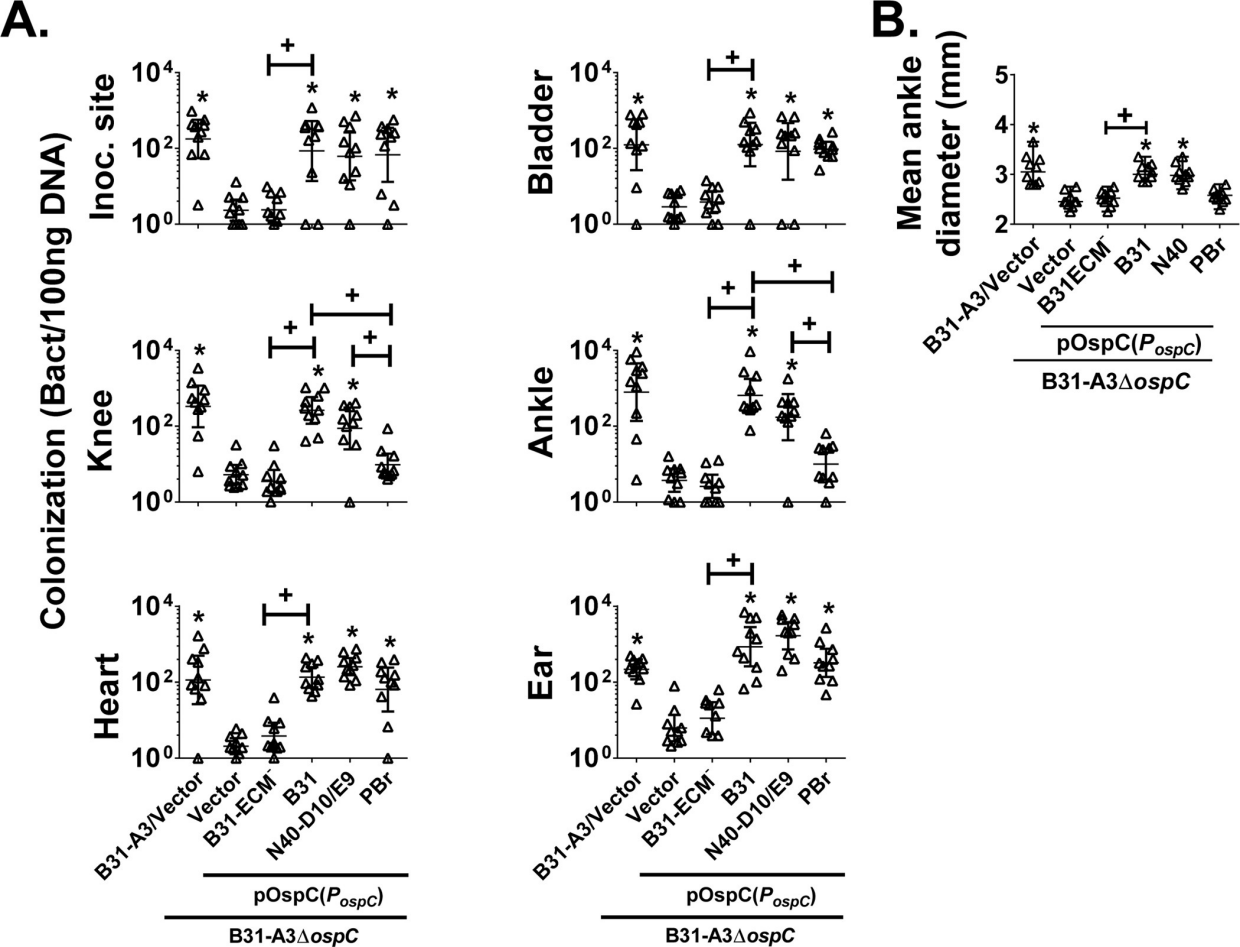
OspC variants promote distinct *B*. *burgdorferi* tissue colonization profiles at 21 days post infection. **(A)**: C3H/HeN mice infected with 10^4^ *B*. *burgdorferi* strain B31-A3/pBSV2G (“B31-A3/Vector”), *ospC-*deficient strain B31-A3Δ*ospC*/pBSV2G (“Vector”), or the deletion strain bearing a plasmid encoding the indicated OspC variants were euthanized at 21 days post infection. The bacterial loads at the inoculation site, ear, bladder, heart, knee, and ankle were determined by qPCR. Data shown are the geometric mean of bacterial loads ± 95% confidence interval of 10 mice per group. Statistical significance was determined using ANOVA with the Kruskal-Wallis test followed by the two-stage step-up method of Benjamini, Krieger and Yekutieli. Significant (*p* < 0.05) differences in spirochete number relative to the *ospC* deletion strain (“*”) and between two strains relative to each other (“+”) are indicated. **(B)** Joint diameter of mice infected with 10^4^ of *B*. *burgdorferi* strain B31-A3/pBSV2G (“B31-A3/Vector”), *ospC*-deficient strain B31-A3Δ*ospC*/pBSV2G (“Vector”), or the deletion strain bearing a plasmid encoding the indicated OspC variants was measured by caliper on day 21 post infection. Data shown are the median of the diameter of ankle joints ± the range of these values in 10 mice per group. Statistical significance was determined using ANOVA with the Kruskal-Wallis test followed by the two-stage step-up method of Benjamini, Krieger and Yekutieli. Significant (*p* < 0.05) differences in the average diameter of ankle joint relative to the *ospC* deletion strain (“*”) and between two strains relative to each other (“+”) are indicated.

To determine if sequence variation of *ospC* results in difference of tissue colonization, C3H/HeN mice were intradermally inoculated with 1x10^4^ *B*. *burgdorferi* strain B31-A3/pBSV2G (Fig 5A, “B31-A3/Vector”), B31-A3Δ*ospC*/pBSV2G (“Vector”), or B31-A3Δ*ospC* producing OspC_B31-ECM_^-^ (“B31-ECM^-^”)_-_, OspC_B31_ (“B31”), OspC_N40-D10/E9_ (“N40-D10-E9”) or OspC_PBr_ (“PBr”). After 21 days, *B*. *burgdorferi* genomes in the inoculation site, bladder, knee, ankle, heart and ear were enumerated by quantitative PCR (qPCR), which amplify this chromosomal gene (Fig 5A). As previously shown [28], mice infected with *ospC*-deficient B31-A3 had significantly (in this case, at least 35-fold) lower bacterial burden in all tissues examined compared to B31-A3/pBSV2G at 21 days post infection (d.p.i.)(Fig 5A, “B31-A3Δ*ospC*/Vector” vs. “B31-A3/Vector"). In fact, in 80–100% of tissue samples, *ospC*-deficient B31-A3 was below the limit of detection of 10 copies per 100ng of DNA. The colonization defect was due to the *ospC* mutation because the introduction of pOspC_B31_ restored bacterial burdens back to wild type levels in all tissues (Fig 5A, “B31”). In addition, we found that B31-A3Δ*ospC* that ectopically produces OspC_N40-D10/E9_, which promotes spirochetal binding to fibronectin in addition to dermatan sulfate (Fig 4), was capable of wild type levels of colonization in all tissues at 21 d.p.i. (Fig 5A, “N40-D10/E9”). The levels of bacteria in all of the tissues tested were 16- to 269-fold greater than that of B31-A3Δ*ospC* harboring vector alone and statistically indistinguishable from that of B31-A3 or B31-A3Δ*ospC*/pOspC_B31_. Importantly, the pBSV2-derived *ospC*-encoding plasmids were retained *in vivo*, because at 21 day post infection, the copy number of these plasmids relative to chromosomal copy number for each of the colonizing strains in the tissues in which they were detected was roughly equivalent to the pBSV2 copy number of spirochetes cultivated *in vitro* with antibiotic selection (S3 and S4 Tables).

Although OspC_PBr_ binds to fibronectin, in contrast to OspC_N40-D10/E9_ and OspC_B31_, it lacks the ability to efficiently bind to dermatan sulfate (Table 1). We found that, 21 days after inoculation, B31-A3Δ*ospC*/pOspC_PBr_ was present at the inoculation site, bladder, heart and ear at levels indistinguishable from wild type B31-A3, indicating that this *B*. *garinii*-derived OspC retained colonization-promoting activity (Fig 5A, “PBr”). However, B31-A3Δ*ospC*/pOspC_PBr_ was not found at high levels in either the knee or ankle at 21 days after inoculation. In both joints, the level of colonization of B31-A3Δ*ospC*/pOspC_PBr_ was at least 64- to 9-fold (and significantly) lower than that promoted by either B31-A3Δ*ospC*/pOspC_B31_ or B31-A3Δ*ospC*/OspC_N40-D10/E9_, respectively, and only 1.8 to 2.7-fold higher than (and statistically indistinguishable from) B31-A3Δ*ospC*. Additionally, B31-A3Δ*ospC*/pOspC_PBr_ could not be detected in 50% and 70% of knee and ankle samples, respectively. These results indicate that OspC_PBr_ promotes colonization of several tissues but not the joints, consistent with the hypothesis that dermatan sulfate binding specifically promotes joint colonization.

To determine whether the inability of OspC_PBr_ to promote long-term colonization of the ankle resulted in a change in a disease manifestation, i.e. joint swelling, we measured the diameters of the ankle joints just prior to euthanasia. Consistent with the lack of joint colonization at 21 days post infection, the diameters of the ankle joints of mice infected with B31-A3Δ*ospC*/pBSV2G (Fig 5B, “Vector”) were significantly smaller than those of mice infected with either the wild type strain B31-A3/pBSV2G (Fig 5B, “B31-A3/Vector”) or B31-A3Δ*ospC*/pOspC_B31_ (Fig 5B, “B31”), both of which colonize this tissue at high levels. Mice infected with B31-A3Δ*ospC*/OspC_N40-D10/E9_ also displayed ankle swelling similar to that caused by strain B31-A3 (Fig 5B, “N40-D10/E9”). Finally, consistent with the lack of detectable joint colonization by B31-A3Δ*ospC*/OspC_PBr_ (Fig 5B, “PBr”), mice infected with these strains had ankle diameters that were not significantly different than those of mice inoculated with the *ospC*-deficient strain B31-A3Δ*ospC*/pBSV2G. Thus, only those OspC proteins that retained the ability to bind to dermatan sulfate (OspC_B31_) or both dermatan sulfate and fibronectin (OspC_N40-D10/E9_) were capable of promoting joint colonization and swelling in mice.

### *B*. *burgdorferi* producing an OspC mutant incapable of binding ECM displays a dramatic colonization defect that is indistinguishable from that of an *ospC*-deficient strain

OspC_B31-ECM_^-^ lacks not only dermatan sulfate-binding activity, but also fibronectin-binding activity. To test whether the ability to promote colonization of any tissue during murine infection correlates with the ability to bind ECM components, we assessed infection by B31-A3Δ*ospC*/pOspC_B31-ECM_^-^. We found that this strain was unable to colonize any of the tissues at levels greater than the *ospC-*deficient mutant (Fig 5A; “B31-ECM^-^”vs. “Vector”). Note that pOspC_B31_ differs from pOspC_B31-ECM_^-^ (as well as from pOspC_N40-D10/E9_ and pOspC_PBr_) not only in the *ospC* coding sequence, but also in the eight nucleotides just 5’ to the *ospC* ATG start codon. Thus, we generated B31-A3Δ*ospC*/pOspC_B31 #2_, which carries the same eight nucleotide substitution 5’ to the *ospC* coding sequence (S4A Fig, “B31-ECM^-^”vs. “B31 #2”). At 21 days post infection, B31-A3Δ*ospC*/pOspC_B31 #2_ but not B31-A3Δ*ospC*/pOspC_B31-ECM_^-^ was capable of colonization. As above, analysis of the relative copy number of pOspC_B31 #2_ showed that the plasmid was retained during murine infection (S5 Table). As expected, a defect in colonization by B31-A3Δ*ospC*/pOspC_B31-ECM_^-^ corresponded to an absence of joint swelling (S4B Fig) or an anti-*B*. *burgdorferi* antibody response any greater than that triggered by a strain entirely lacking *ospC* (S5 Fig). This experiment also showed that the colonization defects of *B*. *burgdorferi* producing OspC_PBr_ or OspC_B31-ECM_^-^ was not associated with differences in antibody response to these strains. Further, the tissue tropism of these strains was not altered upon infection of C3H/HeN-SCID mice, which are incapable of generating an adaptive immune response, indicating that the colonization defects of OspC_PBr_ or OspC_B31-ECM_^-^ did not reflect differences in their ability to either elicit or evade the adaptive immune response (S6 Fig). Instead, these results indicate that the colonization defect of OspC_B31-ECM_^-^ was due to *ospC* coding sequence mutations that abolish ECM binding by this protein.

### ECM-binding by OspC is required for vascular transmigration into joint tissue 24 hours after intravenous inoculation

To determine if OspC plays an essential role in *B*. *burgdorferi* transmigration into knee joint tissue, we used our previously developed 24-hour intravital vascular transmigration assay [62] using *Cd1d*^-/-^ mice. These mice lack iNKT cells, which provide the primary innate immune response in the mouse joint [63, 64]. In the absence of iNKT cells, *B*. *burgdorferi* that transmigrates into joint tissue is not phagocytosed and can be counted one day after *i*.*v*. injection.

**Fig 6:**
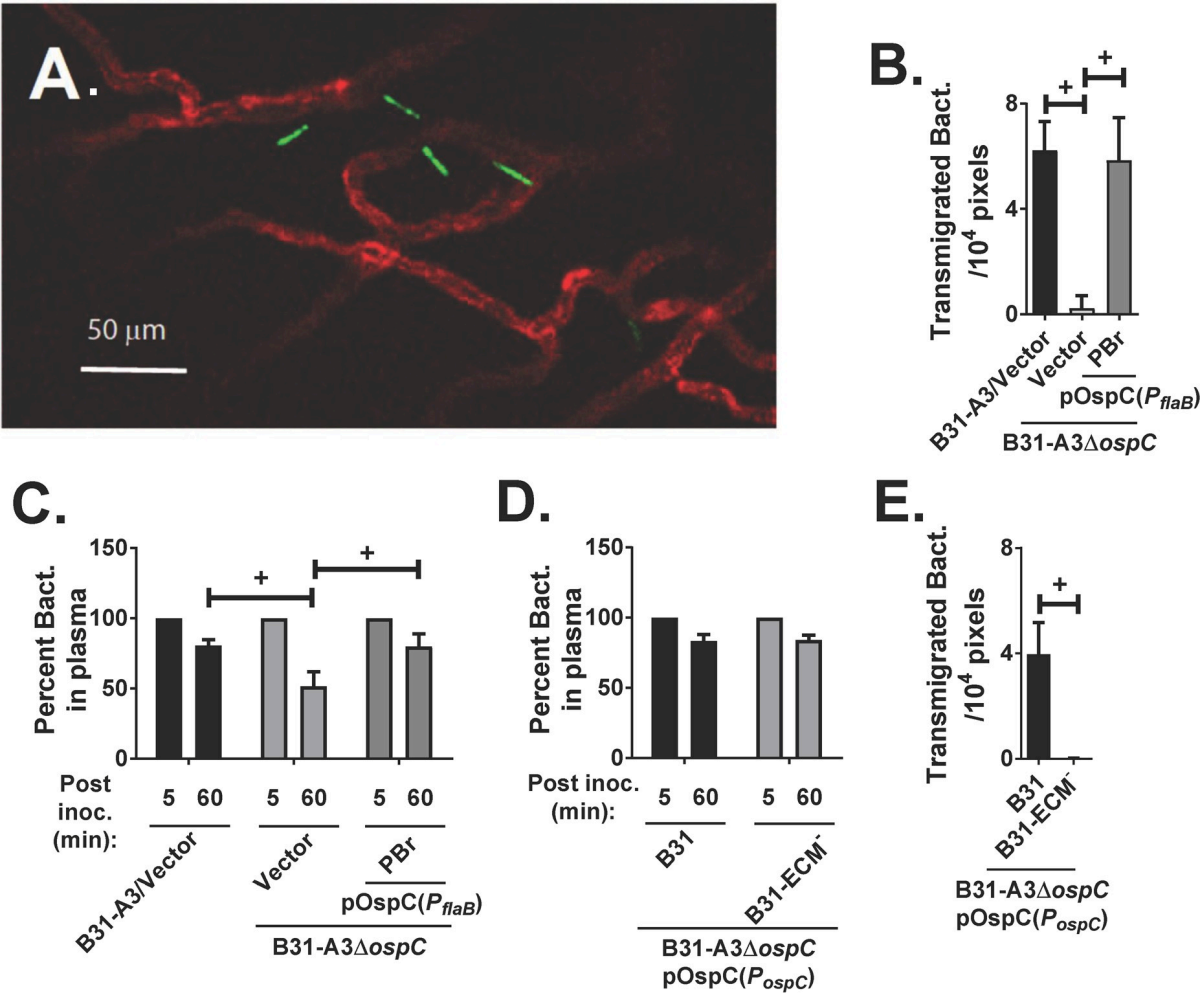
OspC is required for vascular transmigration in the knee of *Cd1d*^-/-^ mice. **(A)** Image of transmigrated GFP-producing *B*. *burgdorferi* by intravital microscopy. Blood vessels were stained with Alexa Fluor 647 anti-PECAM-1 (CD31) antibody shown in red color. **(B)** Transmigration levels of GFP-expressing *B*. *burgdorferi* strains producing wild type OspC_B31_ (“B31-A3/Vector”, i.e. B31-A3/pTM61, GCB 847), no OspC (“Vector”, i.e. B31-A3Δ*ospC*/pTM61spc, GCB 3007) and OspC_PBr_ (“PBr”, i.e. B31-A3Δ*ospC*/pTM61spc-OspC_PBr_(*P*_*flaB*_), GCB 3226). Spirochetes outside of the vasculature (n = 5 mice) were counted in total and plotted as the number of transmigrated spirochetes in an area of 10^4^ pixels. Statistical significance was determined using ANOVA with the Kruskal-Wallis test followed by the two-stage step-up method of Benjamini, Krieger and Yekutieli. Significant (*p* < 0.05) differences in the levels of transmigrated bacteria between two strains relative to each other (“+”) are indicated. **(C)** Clearance of *B*. *burgdorferi* from the vasculature. The change in spirochete concentration between 5 and 60 minutes was determined for each mouse as the percentage of spirochetes present at 60 minutes relative to the initial 5 minute time point. Statistical significance was determined using ANOVA with the Kruskal-Wallis test followed by the two-stage step-up method of Benjamini, Krieger and Yekutieli. Significant (*p* < 0.05) differences in the percent bacteria in plasma between two strains relative to each other (“+”) are indicated **(D)** Spirochete clearance was analyzed for the experiments described in panel E as described in panel C. Statistical significance was determined using ANOVA with the Kruskal-Wallis test followed by the two-stage step-up method of Benjamini, Krieger and Yekutieli. Significant (*p* < 0.05) differences in the levels of transmigrated bacteria between two strains relative to each other (“+”) are indicated. **(E)** Transmigration levels of GFP-expressing *B*. *burgdorferi* strains producing wild type OspC_B31_ (“B31-A3/Vector”, i.e. B31-A3Δ*ospC*/pTM61-OspC_B31_(*P*_*ospC*_), GCB 4458) or OspC_B31-ECM_^-^ (“B31-ECM^-^“, i.e. B31-A3Δ*ospC*/pTM61-OspC_B31_-_ECM_^-^(*P*_*ospC*_), GCB 4452). *Cd1d*^-/-^ mice were injected *via* the tail vein (n = 3 mice) with GFP-producing low-passage strains and transmigration levels were analyzed at 24 hrs as described above.

Mice were injected with *B*. *burgdorferi* strain B31-A3 or an *ospC*-deficient mutant in this strain background harboring the vector pTM61 expressing GFP, or the *ospC*-deficient mutant complemented with a plasmid encoding *ospC* from *B*. *garinii* PBr. The *ospC* coding region in this complementing plasmid was under the control of the *flaB* promoter (see S1 Table for strain information). Intravital microscopy using a spinning disk laser confocal microscope was performed at 24 hrs post-inoculation. Upon staining blood vessels with Alexa Fluor 647 anti-PECAM-1 antibody, images of the left knee to a depth of 200 μm were collected during the one hr intravital experiment. Spirochetes that had transmigrated into the tissue surrounding the knee were counted (Fig 6A). Compared with the wild type strain, a dramatic decrease in transmigration into the joint tissue was observed for the *ospC*-deficient strain. In spite of the fact that OspC_PBr_ did not promote joint localization in the short-term infection model or joint colonization model, we found that complementation of the *ospC*-deficient strain with a plasmid that produces OspC_PBr_ restored vascular transmigration (Fig 6B, “PBr”).

A potential confounding factor in this experiment is the ability of OspC, by binding the complement component C4b, to promote bloodstream survival [43]. Indeed, we found here that the rate of clearance of the *ospC*-deficient spirochete was significantly faster compared with the wild type strain, and that complementation with an OspC_PBr_–producing plasmid restored the wild type clearance rate in *Cd1d*^-/-^ mice (Fig 6C). Thus, OspC could be directly involved in spirochete extravasation or, by protecting *B*. *burgdorferi* from clearance, indirectly promoting detectable levels of transmigration. Therefore, we assessed the transmigration of the OspC_B31-ECM_^-^ mutant, which we showed retains the abilty to bind C4b (S2D Fig). In contrast to the *ospC*-deficient *B*. *burgdorferi*, the strain producing OspC_B31-ECM_^-^ displayed a clearance rate almost identical with wild type *B*. *burgdorferi* strain B31-A3 harboring the vector (Fig 6D). Moreover, compared with wild type *ospC*_B31_, the OspC_B31-ECM_^-^ mutant was entirely defective at transmigration into the knee (Fig 6E). Therefore, we conclude that OspC, in addition to protection from clearance, plays an essential role in *B*. *burgdorferi* vascular transmigration into joint tissue.

## Discussion

Tissue colonization by multiple bacterial pathogens is a complex process that includes bloodstream survival, distinct and increasingly stable interactions with the vascular wall, and tissue invasion [8, 12]. *B*. *burgdorferi* OspC lipoprotein is upregulated while the bacteria are in the midgut of a feeding tick and is essential for the early stages of mammalian function [26, 28, 65, 66]. In addition, OspC is highly variable and distinct variants are associated with differences in strain invasiveness [32, 67, 68]. These observations have prompted several studies of its potential role in mammalian colonization [41–43, 69]. We recently found that OspC, by binding the complement component C4b, promotes bloodstream survival at early stages of infection [43]. Given that OspC of *B*. *burgdorferi* strain N40-D10/E9 was enriched for tissue localization upon i.v. inoculation of a phage display library [49], here we examined the role of OspC as a potential adhesin that influences tissue colonization by Lyme disease bacteria. We found that recombinant OspC_N40-D10/E9_ binds to both dermatan sulfate and fibronectin and identified three functional OspC groups, typified by: (1) OspC_N40-D10/E9_, which efficiently (i.e., *K*_D_ ≤ 0.8 μM by SPR) binds dermatan sulfate and fibronectin; (2) OspC_B31_, which binds dermatan sulfate but not fibronectin; and (3) OspC_PBr_, which binds fibronectin but not dermatan sulfate. Using a one-hour *i*.*v*. C3H mouse infection model that likely measures initial vessel wall adherence in different vascular beds, we found that OspC_N40-D10/E9_ and OspC_B31_, which both bind dermatan sulfate, were capable of promoting spirochetal localization to ankles when produced on the surface of an *ospC*-deficient *B*. *burgdorferi* strain. In contrast, OspC_PBr_, which binds fibronectin but not dermatan sulfate, did not.

To test whether the fibronectin and dermatan sulfate adhesive activities of OspC are essential for long-term colonization, we first compared the sequences of OspC variants capable or incapable of binding dermatan sulfate to identify a highly basic candidate GAG-binding region of α-helix 3 and loop 4, conserved in all of the OspC-displaying phage that promoted tissue localization in mice. Methionine substitution of five lysine residues in this region resulted in OspC_B31-ECM_^-^, which retains secondary structure but is incapable of recognizing either dermatan sulfate or fibronectin. We then tested isogenic strains that produce either OspC_B31_ or OspC_B31-ECM_^-^ for the ability to colonize C3H mice after *i*.*d*. inoculation. We found that whereas wild type OspC_B31_ promoted colonization of all tissues tested at three weeks post infection, the strain producing OspC_B31-ECM_^-^ was no more capable of colonization of any tissues than the control *ospC*-deficient strain. These results indicate that not only does OspC promote spirochetal binding to the ECM components, fibronectin or dermatan sulfate, but this adhesive activity is essential for mammalian infection.

Given that OspC displays strain-specific differences, we evaluated the role of such differences in long-term colonization of mammalian tissues. Upon *i*.*d*. inoculation of C3H mice with isogenic *B*. *burgdorferi* strains that differ only in their *ospC* coding sequence, we found that the OspC_N40-D10/E9_, OspC_B31_, and OspC_PBr_, each of which binds fibronectin, dermatan sulfate, or both, were fully capable of promoting colonization of the injection site, ear, heart and bladder at three weeks post infection. However, only those variants capable of binding dermatan sulfate, i.e. OspC_N40-D10/E9_ and OspC_B31_, were able to promote colonization of the knee and ankle joints; joint colonization by the strain producing OspC_PBr_ was no greater than an *ospC*-deficient mutant. These results suggest that dermatan sulfate binding by OspC is required for joint colonization.

Lyme disease spirochetes encode a plethora of GAG-binding adhesins, including BBK32, DbpA and DbpB, which bind dermatan sulfate, Lmp1 and Bgp, which bind chondroitin-6-sulfate, and OspF protein family members, which bind heparan sulfate [54–57, 59, 70]. The dermatan sulfate-binding activity of DbpA is essential for colonization of multiple tissues [21, 22] whereas that activity of BBK32 promotes joint colonization [20]. *B*. *burgdorferi* producing mutants of OspC, BBK32 or DbpA specifically defective for dermatan sulfate binding demonstrate colonization defects, indicating that these GAG-binding activities, which are of roughly comparable affinity (*K*_D_s of 0.21 to 0.91 μM) when evaluated with purified substrates [20, 22], function in a non-redundant fashion during colonization. Presumably, this non-redundancy reflects differences in adhesin-mediated GAG-binding activities that have not yet been discerned and a tissue colonization process that requires the sequential or simultaneous action of each of these activities.

The association of strain invasiveness with *ospC* type could reflect direct involvement of the OspC protein in tissue tropism, or could simply be a genetic marker of spirochetal strains that encode non-*ospC* tropism determinants [67, 68]. Our analyses of isogenic strains revealed that the OspC variant produced by a Lyme disease strain is in fact a direct determinant of the strain’s tissue tropism; in fact variants derived from *B*. *burgdorferi* sensu stricto, a species that is associated with human Lyme arthritis [3], promote greater joint colonization and more severe disease in the C3H mouse than variants produced by ‘non-arthritogenic’ species of Lyme spirochete. An attractive model is that a complex spectrum of GAG-binding activities conferred by variable adhesins such as OspC, DbpA or members of the OspF family contributes to observed differences in tissue tropism by different strains during human Lyme disease [22, 56]. Of note, OspC is a critical determinant of the early stage infectivity of *B*. *burgdorferi*, when the bacteria are establishing infection and actively disseminating from the site of the tick bite.

Dermatan sulfate GAGs are found in the vascular bed of joints [71]. The ability of OspC_N40-D10/E9_ and OspC_B31_ to foster localization to ankles 60 minutes after *i*.*v*. inoculation suggests that these dermatan sulfate-binding proteins promote initial interaction with joint vasculature. The finding that OspC_N40-D10/E9_ and OspC_B31_ each promoted joint colonization three weeks after i.d. inoculation raised the possibility of a function in tissue invasion subsequent to early vascular attachment. Intravital microscopy was used to quantify joint invasion 24 h after i.v. infection of *Cd1d*^-/-^ BALB/c mice with GFP-producing strains provided a direct means to interrogate a role for OspC in joint tissue invasion. We found that a strain producing OspC_PBr_ enters joints far more efficiently than an *ospC*-deficient strain. This result was not predicted from our finding that this variant was not capable of promoting joint localization one hr after i.v. infection or joint colonization three weeks after i.d. inoculation and suggests the possibility that although OspC_PBr_ is fully functional in promoting vascular transmigration, it may nonetheless be compromised in an earlier, presumably distinct step of the dissemination cascade [72–74] [75]. However, direct comparison of the three types of murine infection assays is confounded by differences in several experimental parameters, including *B*. *burgdorferi* and murine strain background, control of *ospC* expression, and route and duration of infection. Moreover, assessing whether OspC_PBr_ directly participated in the movement of bacteria from the bloodstream into the joint was complicated by the fact that this protein, as predicted by previous studies [18, 43], enhanced spirochetemia in *Cd1d*^-/-^ BALB/c mice. Thus, we instead compared isogenic strains that produced either OspC_B31_, which binds dermatan sulfate, or OspC_B31-ECM_^-^, which does not. OspC_B31-ECM_^-^ retained the ability to bind C4b and promoted bloodstream survival in *Cd1d*-deficient mice indistinguishably from OspC_B31_, allowing a straightforward comparison of joint invasion promoted by the two proteins. This comparison revealed a direct role of the OspC adhesive activity in joint invasion.

Combined with previous work defining the role of several *B*. *burgdorferi* adhesins in the tissue invasion process, an emerging model is that movement of the spirochete from the bloodstream into tissue is a multistep process mediated by a series of adhesive events [8, 12]. The fibronectin-binding activity of BBK32 promotes an initial ‘tethering’ interaction with the vascular wall through catch-bond interactions under shear stress, and its dermatan sulfate-binding activity promotes the more stable ‘dragging’ interaction [9, 50, 76, 77]. Thereafter, integrin-binding by the *B*. *burgdorferi* outer membrane protein P66 is essential for joint invasion [62]. Here we showed a critical role for dermatan sulfate-binding by OspC for vascular transmigration. As is the case for P66-endothelial interactions, the interaction of OspC with the vascular endothelium leading to extravasation appears to occur subsequent to early tethering and dragging interactions that decelerate the spirochetes under the shear stress of bloodflow, because the presence of OspC does not impart detectable tethering or dragging interactions in the absence of both BBK32 and VlsE, powerful adhesins that promote deceleration under shear flow [Tan, X. et al, manuscript in preparation]. The molecular mechanism by which OspC promotes vascular transmigration remains unknown at this time, but it is tempting to speculate that it may induce endothelial signaling to promote cellular changes required for spirochetal transmigration. Nonetheless, a direct role of OspC in transmigration subsequent to deceleration certainly does not preclude earlier role(s) in survival and the tissue colonization pathway. The emerging picture is that OspC is a multifunctional protein with crucial roles in the pathogenesis process.

Invasion of mammalian leukocytes into tissues during the inflammatory process features several parallels, as it is well-documented to occur via a multi-step process involving counterreceptors for both lectins and integrins [78, 79]. Given these conserved themes, it seems likely that the observed tropism of other species of Lyme disease for non-joint tissues such as skin, heart or neural tissue may result from the sequential action of alternate sets of bacterium-host cell interactions. Similar multi-faceted analyses of isogenic strains of such Lyme disease species that vary in their repertoire of adhesive surface factors, such as the study described here, is an important goal. With the with further development of genetic techniques in these species, as well as the development of small animal models that reflect human tropism, such studies will provide rigorous means to identify and characterize these interactions and to better understand the molecular basis underlying diverse tissue tropisms characteristic of different Lyme disease spirochetes.

## Materials and methods

### Ethics statement

All mouse experiments were performed in strict accordance with all provisions of the Animal Welfare Act, the Guide for the Care and Use of Laboratory Animals, and the PHS Policy on Humane Care and Use of Laboratory Animals. The protocol was approved by the Institutional Animal Care and Use Committee (IACUC) of Medical College of Wisconsin, Tufts University School of Medicine, Wadsworth Center-New York State Department of Health, or University of Calgary. All efforts were made to minimize animal suffering.

### Animals

Female C3H/HeN or C3H/HeN-SCID mice were acquired from Charles River Laboratories (Charles River Laboratories International, Inc., Wilmington, MA) and used at 4 wks of age for long term intradermal inoculation studies, or 8 wks of age or older for short term intravenous (i.v) inoculation experiments. For intravital microscopy *Cd1d*^-/-^ mice in a BALB/c background (Jax #2962) were bred in-house at the Clara Christie Centre for Mouse Genomics at the University of Calgary. Mice of both genders between 6–8 weeks of age were used.

### Bacterial strains and culture conditions

All strains used in this study are described in S1 Table. *B*. *burgdorferi* strains were grown in Barbour-Stoenner-Kelly (BSKII) medium made in-house [80] containing 6% rabbit serum at 33°C to a density of 1x10^8^ spirochetes/ml. When antibiotic selection was required, kanamycin was used at 200 μg/ml and gentamicin was used at 40 μg/ml. Presence of genomic plasmids and shuttle vectors was confirmed in each culture by PCR prior to inoculation into mice (S2 Table) [81–83]. *Escherichia coli* strains DH10B, BL21 and their derivatives (S1 Table) were grown in Luria-Bertani broth (BD Bioscience, Franklin Lakes, NJ) or agar supplemented with ampicillin (100 μg/ml) when necessary.

### Strain construction

The *ospC*-deficient *B*. *burgdorferi* strain B31-A3 (OspCK1), generated previously [28], was engineered to express o*spC* alleles from various strains and species of *Borrelia* from the *ospC* promoter and ribosome binding site from strain B31-A3, or constitutively from the flagellar promoter (*flaB*) and ribosome binding site native to each *ospC* allele [43], in pBSV2G (S1 Table). This strain as background in this study because of the available genetic tools and information (plasmid profiles) and small animal models that can apply to this strain. To express *ospC*_*B31*_ under control of the OspC_B31-A3_ promoter, 200bp upstream of the start codon of *ospC*_*B31-A3*_ with the coding sequences of *ospC*_*B31*_ were cloned into pBSV2G, resulting in pOspC_B31_ [28]. For expression of *ospC*_*N40-D10/E9*_, *ospC*_*PBr*_, or *ospC*_*B31-ECM*_^-^ under control of the *ospC*_B31-A3_ promoter, *ospC* alleles were amplified using primers (S2 Table) containing SalI and BamHI sites at the 5’and 3’ ends, respectively, from genomic DNA isolated from *B*. *burgdorferi* strains B31-A3 and N40-D10/E9, and *B*. *garinii* strain PBr using the Wizard SV Genomic DNA Purification System (Promega corp., Madison, WI). Amplified DNA fragments were inserted into the TA cloning vector, pGEM-T Easy (Promega corp.). To generate pGEM-T Easy encoding OspC_B31-ECM_^-^, site-directed, ligase-independent mutagenesis (SLIM) was used to replace lysines-116, 121, 123, 128, and 129 of OspC from *B*. *burgdorferi* strain B31-A3 with methionines on pCR2.1 TOPO encoding OspC_B31-A3_ as described [84]. Those pGEM-T Easy-derived plasmids encoding *ospC*_*N40-D10/E9*_ or *ospC*_*PBr*_, *ospC*_*B31-ECM*_^-^, were then digested with SalI and BamHI and *ospC* alleles were subcloned into pBSV2G at the SalI and BamHI sites [85]. The promoter region of *ospC* from *B*. *burgdorferi* B31-A3, 184 bp to 9 bp upstream of the start codon of *ospC* [54], was also PCR amplified, adding HindIII and SalI sites at the 5’and 3’ ends, respectively, using primers pospCfp and pospCrp (S2 Table), resulting in plasmids pOspC_N40-D10/E9_, pOspC_PBr_, and pOspC_B31-ECM_^-^. Note that pOspC_B31_ differs from pOspC_B31-ECM_^-^ (as well as from pOspC_N40-D10/E9_ and pOspC_PBr_) not only in the *ospC* coding sequence, but also in the eight nucleotides just 5’ to the *ospC* ATG start codon. We thus also generated pOspC_B31 #2_ with the same nucleotides 5’ to the *ospC* ATG start codon (S1 Text).

To generate strains to be utilized in short-term *i*.*v*. infections, genomic DNA was isolated from *B*. *burgdorferi* strain B31-A3 and N40-D10/E9, and *B*. *garinii* strain PBr using the Wizard SV Genomic DNA Purification System (Promega corp., Madison, WI). *ospC* alleles containing the native ribosome binding sites (which were identical) were amplified from genomic DNA using the oligonucleotides described in S2 Table. PCR fragments containing the *ospC* alleles were cloned in pGEM-T Easy (Promega corp., Madison, WI). Plasmid pTM61spc-MCS was generated by annealing oMCS3 and oMCS4 and ligating the fragment into pGEM-T Easy. The MCS was excised from pGEM-T Easy (Promega corp., Madison, WI) using *Bsp*HI and *Ear*I, and ligated in pTM61spc [62] at the BspHI site. The flagellar promoter was amplified from *B*. *burgdorferi* strain B31-A3 genomic DNA using P-FlaB oligonucleotides (S2 Table), ligated in pGEM-T Easy (Promega corp., Madison, WI), excised using NheI and SalI (NEB, Ipswich, MA), and subcloned in the NheI and XhoI sites of pTM61spc-MCS (S1 Table). The *ospC* alleles in pGEM-T Easy were excised from the vector using SacI and XmnI (New England BioLabs, Inc., Ipswich, MA), and cloned downstream of the *flaB* promoter in pTM61spc-MCS (S1 Table). The *flaB* promoter-*ospC* allele fragments were excised from pTM61spc-MCS using *Hind*III and *Bam*HI and ligated in pBSV2G [28] at the HindIII/BamHI sites.

To generate GFP-producing *B*. *burgdorferi* strains, the *ospC*-deficient *B*. *burgdorferi* strain B31-A3Δ*ospC* (OspCK1) [28] was engineered to express *ospC* alleles under the control of the constitutive flagellar promoter (*P*_*flaB*_) in pTM61spc-MCS as previously described [43] or under the control of the native *ospC* promoter in pBSV2G (S1 Table). To build the complementing strains GCB4458 (B31-A3Δ*ospC*/pTM61-OspC_B31_(*P*_*ospC*_)) and GCB4452 (B31-A3Δ*ospC*/pTM61-OspC_B31-ECM_^-^(*P*_*ospC*_)) with *ospC* under the control of the *ospC* promoter in pTM61, the wild type (*ospC*_*B31*_) and mutated (*ospC*_B31-ECM_^-^) genes were first sub-cloned in pJET1.2 (ThermoScientific, Waltham, MA). To do so, both genes were amplified by using OspC_B31_fp/pTM61 and OspC_B31_rp/pTM61, including restriction sites for SbfI and AvrII respectively (S2 Table). The DNA used as template for the wild type *ospC*_B31_ under the control of the *ospC* promoter was pOspC_B31_(*P*_*ospC*_), while *ospC*_B31-ECM_^-^ controlled by the *ospC* promoter was amplified from pOspC_B31-ECM_^-^(*P*_*ospC*_). Each insert was ligated into pJET1.2.Plasmids pTX4 (pJET::*ospC*_B31_), pTX5 (pJET::*ospC*_B31-ECM_^-^) and pTM61 were double digested with the restriction enzymes SbfI and AvrII, acquired from NEB. Digestions were run in a 1% agarose gel and the bands corresponding to pTM61 (6.7 Kb), *ospC*_B31_ (817bp) and *ospC*_B31-ECM_^-^ (817bp) were gel purified. Ligations were performed to obtain plasmids pMC114 (pTM61::*ospC*_B31_) and pMC115 (pTM61::*ospC*_B31-ECM_^-^). The resulting constructs were sequenced with oligos OspC_B31_fp/pTM61 and OspC_B31_rp/pTM61 to confirm their integrity. Strains GCE3815 (*ospC*_B31_) and GCE3817 (*ospC*_B31-ECM_^-^) were grown overnight in LB to purify the respective plasmids pMC114 and pMC115 by midiprep (Qiagen, Germantown, MD). 150 μg of plasmids pMC114 and pMC115 were incubated individually with the GpC methyltransferase M.CviPI according to NEB protocols. After precipitation with isopropanol followed by 70% ethanol, 50 μg of each plasmid was transformed into electrocompetent strain B31-A3Δ*ospC* (GCB3022). Candidate transformants were screened for the presence of the gentamicin cassette and the *ospC* insert, either wild type (*ospC*_*B31*_) or ECM mutant (*ospC*_B31-ECM_^-^). They were also tested for green fluorescence (GFP harbored in pTM61). Finally, the plasmid profiles were analyzed as reported [81]. The strain complemented with the wild type *ospC* version lost lp28-4 and lp56. However, these plasmids have not been shownto affect infectivity [83]. The final strains (S1 Table) were GCB4458 (B31-A3Δ*ospC*/pTM61-OspC_B31_(*P*_*ospC*_)) and B31-A3Δ*ospC*/pTM61-OspC_B31-ECM_^-^(*P*_*ospC*_) (GCB4452).

Plasmid constructs in *E*. *coli* were isolated using the QIAGEN Plasmid Maxi Kit (Qiagen, Valencia, CA) and transformed into *B*. *burgdorferi* by electroporation of 50 μg of CpG methylated DNA and plating into semi-solid agar or in liquid in 96-well plates as previously described [24, 86]. All *ospC* alleles were found to have the correct sequence when carried on pBSV2G or pTM61 in *B*. *burgdorferi* with the exception of the *ospC* allele from *B*. *burgdorferi* strain N40-D10/E9, which was found to encode the single missense mutation N46S after multiple independent PCR amplifications [43]. The quantitation of surface exposure of the resulting strains is described in S1 text.

### Recombinant GST-fusion proteins

To generate recombinant GST-tagged OspC proteins, the *ospC* open reading frames lacking the putative cleaved signal sequences from *B*. *burgdorferi* strains B31-A3, 297, and N40-D10/E9, *B*. *garinii* strain PBr, *B*. *afzelii* strain VS461, and an altered open reading frame encoding OspC_B31-ECM_^-^ (lysine residues 116, 121, 123, 128, and 129 were replaced with methionines) were amplified (S2 Table) and cloned into pGEX4T2 at the BamHI and SalI sites (GE Healthcare, Piscataway, NJ), as previously described [24, 43]. Plasmids were transformed into *E*. *coli* strain BL21(DE3) and the plasmid inserts were sequenced (Tufts core sequencing facility). The GST-tagged OspC variants were produced and purified by glutathione chromatography according to the manufacturer’s instructions (BD Bioscience, Franklin Lakes, NJ).

### Glycosaminoglycan and fibronectin binding assays

Quantitative ELISA for fibronectin and dermatan sulfate binding by OspC proteins was performed as previously described [87] with the following adjustments. To coat the wells with fibronectin and dermatan sulfate, these wells were incubated with 10 μg/ml of purified fibronectin or dermatan sulfate in the coating buffer (0.05 M Na_2_CO_3_, pH 9.0). One hundred microliters of increasing concentrations (0.03125, 0.0625, 0.125, 0.25, 0.5, 1, 2 μM) of GST (negative control) or GST-tagged OspC variants, including OspC_B31_, OspC_297_, OspC_N40-D10/E9_, OspC_VS461_, OspC_PBr_, or OspC_B31-ECM_^-^ were added to the coated wells and incubated for 1 hr at ambient temperature. To detect the binding of GST-tagged proteins, goat anti-GST (Sigma-Aldrich, St. Louis, MO; 1:200) and HRP-conjugated donkey anti-goat IgG (Promega, Fitchburg, WI; 1:1,000) were used as primary and secondary antibodies, respectively. The plates were washed three times with PBST (0.05% Tween20 in PBS buffer), and 100 μl of tetramethyl benzidine (TMB) solution (KPL, Gaithersburg, MD) was added to each well. The reaction was stopped by adding 100 μl of 0.5% hydrosulfuric acid to each well and plates were read at 405 nm using a Synergy HT ELISA plate reader (BioTek, Winooski, VT). To determine the dissociation constant (*K*_D_), the data were fit to the following equation using KaleidaGraph software (Version 4.1.1 Synergy Software, Reading, PA).

$$OD405=\frac{OD405max\left[OspC\;proteins\right]}{K_{D}\;\left[OspC\;proteins\right]}$$

### Surface Plasmon Resonance (SPR)

Interactions of OspC with fibronectin or dermatan sulfate were analyzed by SPR using the Biacore 3000 (GE Healthcare, Piscataway, NJ). To conjugate SPR chips with fibronectin or dermatan sulfate, 10 μg of fibronectin in 10 μl of acetate buffer (CH_3_COONa, pH 4.0) was flowed through a CM5 chip (GE Healthcare, Piscataway, NJ) whereas 10 μg of dermatan sulfate in 10 μl of PBS buffer was biotinylated as described [24] and flowed through an SA chip (GE Healthcare). A control flow cell was injected with PBS buffer without fibronectin or dermatan sulfate. For quantitative SPR experiments to determine fibronectin- or dermatan sulfate–binding, 10 μl of increasing concentrations (0, 15.625, 31.25, 62.5, 125, 250, 500 nM) of OspC variants including OspC_B31_, OspC_297_, OspC_N40-D10/E9_, OspC_VS461_, OspC_PBr_, or OspC_B31-ECM_^-^, were injected into the control cell followed by the flow cell with immobilized fibronectin or dermatan sulfate at 10 μl/min, 25°C. To obtain the kinetic parameters of the interaction, mean sensogram data were fit to a curve using BIAevaluation software version 3.0 (GE Healthcare, Piscataway, NJ) with the one step biomolecular association reaction model (1:1 Langmuir model), resulting in optimum mathematical fit with the lowest Chi square values.

### Binding of radiolabeled *B*. *burgdorferi* to purified fibronectin and dermatan sulfate

Binding of *B*. *burgdorferi* to purified fibronectin or dermatan sulfate was determined essentially as previously described [24]. Briefly, spirochetes were radiolabeled with [^35^S] methionine, and 1 x 10^8^ radiolabeled bacteria were added to microtiter plate wells previously incubated with 250 μg/ml purified fibronectin, dermatan sulfate or BSA (negative control). After 1 hr at 37°C, unbound bacteria were removed by washing with PBS containing 0.2% BSA. Plates were air-dried, and bound bacteria in each well were quantified by liquid scintillation. The percent of bound bacteria was calculated by normalizing the counts of each well to the counts in the inoculum.

### Mouse infections

Four-week-old female C3H/HeN or C3H/HeN-SCID mice were infected by intradermal injection with 1X10^4^ *B*. *burgdorferi* strain B31-A3/pBSV2G, an *ospC*-deficient *B*. *burgdorferi* strain B31-A3Δ*ospC*/pBSV2G, or B31-A3Δ*ospC* carrying a plasmid encoding *ospC*_B31_, *ospC*_N40-D10/E9_, *ospC*_PBr_, or *ospC*_B31-ECM_^-^ driven by the B31-A3 *ospC* promoter (S2 Table). Mice were euthanized at 21 d.p.i. and the skin at the inoculation site, knee, ankle, bladder, heart, and ear were isolated. DNA was extracted from these tissues using the DNeasy Blood & Tissue kit (Qiagen, Valencia, CA). *B*. *burgdorferi* genomes in each tissue were quantified by qPCR (S2 Table) using a CFX Connect Real-Time PCR detection system (BioRad, Hercules, CA) in conjunction with SYBR green PCR Mastermix (BioRad, Hercules, CA) (95.0°C for 5min, 94.0°C for 1 sec, 66°C for 15 sec repeated 45 times) [88]. The number of *recA* copies was calculated by first establishing a Ct standard curve using known amounts of the *recA* gene extracted from *B*. *burgdorferi* strain B31-A3. Genome numbers in each sample were derived by comparison to the standard curve.

The short term *i*.*v*. inoculation experiments were performed as described [18, 43]. C3H/HeN mice under anesthesia were inoculated retro-orbitally with 1x10^8^ *B*. *burgdorferi* cells in 100 μl. After 1 hr, blood was collected by cardiac puncture, and the mice were perfused with sterile saline to wash away any unbound bacteria from the vasculature. Tissues were harvested and DNA isolated for quantitation of *Borrelia* and mouse genomes by qPCR [18, 43].

### Evaluation of joint swelling

C3H/HeN mice were intradermally inoculated with 1X10^4^ *B*. *burgdorferi* strain B31-A3 carrying the empty vector, B31-A3Δ*ospC* carrying the empty vector, or *ospC*_*B31*_, *ospC*_*N40-D10/E9*_, *ospC*_*PBr*_, *or ospC*_*B31-ECM*_^*—*^complemented strains. Mice were examined for swelling of the ankles on 21 d.p.i. as described previously [89]. Ankles from the rear legs of each mouse were measured using a high precision metric caliper in a blinded fashion. The thickest diameters of the ankles were measured in each mouse, and the data are presented as the median diameter of ankles ± the range, n = 10.

### Intravital vascular transmigration assays

For vascular transmigration assays, the deeply anesthetized *Cd1d*^-/-^ mice were intravenously inoculated with spirochetes 24 hrs before imaging as described previously [62]. A GFP-producing low-passage, infectious, *B*. *burgdorferi* strain was injected into the tail vein of *Cd1d*^-/-^ mice (4 x 10^8^ spirochetes per mouse). At 24 hrs post spirochete injection, vascular transmigration was scored in the knee to a depth of about 150 microns in the living mice by intravital microscopy using a spinning disk laser confocal microscope. The area exposed was over the patellar ligament and to the medial side of the right hind limb. We examined post-capillary venules that drain blood from the anterior tibialis muscle and joint with the anterior tibial vein that drains into the great saphenous vein (see [62] for further details). Blood vessels were stained with Alexa Fluor 647 anti-PECAM-1 (CD31) antibody indicated in red color. The total spirochete number and the surgical area were determined using Adobe Photoshop CC (San Jose, CA) for calculation of the number of spirochetes per area (10^4^ pixels). For spirochete clearance assays on mice used for intravital microscopy, blood was withdrawn from mice at 5 and 60 minutes post-inoculation. Blood cells were allowed to settle overnight in a heparinized capillary tube and spirochetes in the plasma were directly counted by dark-field microscopy [62].

### Statistical analysis

Statistical analyses were performed using the Mann-Whitney unpaired t-test or ANOVA using the Kruskal-Wallis test followed by the two-stage step-up method of Benjamini, Krieger and Yekutieli using GraphPad Prism 7 as indicated in each figure legend. All qPCR data were log transformed prior to analysis. A P-value < 0.05 was considered significant. The data represent the median ± range, mean ± standard deviation, mean ± SEM, geometric mean ± 95% confidence interval, or geometric mean ± geometric standard deviation as indicated in each figure.

## Supporting information

S1 Fig OspC class protein sequence alignment. ClustalW alignment performed on protein sequences of OspC from *B*. *burgdorferi* (Bb) strain B31 (“Bb B31”), 297 (“Bb 297”), and N40-D10/E9 (“Bb N40-D10/E9”), *B*. *afzelii* strain VS461 (“Ba VS461”), and *B*. *garinii* strain PBr (“Bg PBr”) using BioEditor Sequence Alignment Editor [90]. At each position, conserved amino acids are highlighted in black and similar amino acids are highlighted in grey. Asterisks indicate OspC_B31_ lysines that were mutated to methionines to abrogate ECM binding, resulting in OspC_B31-ECM_^-^. The fragment of OspC from N40-D10/E9 selected for binding to joint and heart by phage display [49] is underlined.
(TIF)

S2 Fig Mutation of lysine residues 116, 121, 123, 128, and 129 of OspC_B31_ does not affect its structure or ability to bind C4b. (A) The location of the quintuple basic residues (K116, K121, K123, K128, K129) mutated in OspC_ECM_^-^ mapped onto the crystal structure of OspC_B31_ [91, 92]. Helix 3 and loop 4 are highlighted in blue and green, respectively. (B) Amino acid sequence alignment of helix 3 and loop 4 of different *ospC* alleles. “*” indicates residues mutated in OspC_B31-ECM_^-^. (C) Far-UV CD analysis of OspC_B31_ and OspC_B31-ECM_^-^. The molar ellipticity, Φ, was measured from 190 to 250 nm for 10 μM of each protein in Tris buffer (pH7.5). (D) The indicated concentrations of GST, GST-OspC_B31_, or GST-OspC_B31-ECM_^-^ were added to quadruplicate wells coated with human C4b, and binding (± standard deviation) was measured by ELISA (see Experimental Procedures). Shown is a representative of three independent experiments. The K_D_ values obtained from the average of three independent experiments were calculated and shown in the inset.
(TIF)

S3 Fig OspC variants are produced on the surface of an infectious *B*. *burgdorferi* strain. **(A)** Flow cytometry analysis of OspC localized to the surface of parental strain *B*. *burgdorferi* B31-A3/pBSV2G (“B31-A3/Vector”; red), *ospC* deletion strain B31-A3Δ*ospC*/pBSV2G (“B31-A3Δ*ospC*/Vector”; orange), and the *ospC* deletion strain bearing a plasmid encoding OspC_B31_ (“B31-A3Δ*ospC*/pOspC_B31_”; blue). **(B)** OspC (top) or flagellin (bottom) on the surface of the indicated untreated (solid bars) or methanol-permeabilized (open bars) strains was quantitated by flow cytometry after staining with anti-OspC or anti-flagellin, respectively (see Materials and Methods). Values shown are relative to the production levels of OspC or flagellin on the surface of permeabilized *B*. *burgdorferi* strain B31-A3 harboring the empty vector. Each bar represents the mean of four independent determinations ± SEM. (*): indicates that surface production of the indicated proteins was significantly lower (* = P < 0.05, ANOVA with the Kruskal-Wallis test followed by the two-stage step-up method of Benjamini, Krieger and Yekutieli) than the detected production of OspC or Flagellin by *B*. *burgdorferi* strain B31-A3 harboring the vector.
(TIF)

S4 Fig The colonization defect of OspC-ECM^-^ is due to the alteration of coding sequence. **(A)** C3H/HeN mice infected with 10^4^ *B*. *burgdorferi* strain B31-A3/pBSV2G (“B31-A3/Vector”), *ospC* deletion strain B31-A3Δ*ospC*/pBSV2G (“Vector”), or the deletion strain bearing a plasmid encoding either OspC_B31-ECM_- or OspC_B31 #2_, which differ only in the *ospC* coding sequence, were sacrificed at 21 days post infection. The bacterial loads at the inoculation site, ear, bladder, heart, knee, and ankle joint were determined by qPCR. Data shown are the geometric mean of bacterial loads ± 95% confidence interval of 10 mice per group. Statistical significance was determined using ANOVA with the Kruskal-Wallis test followed by the two-stage step-up method of Benjamini, Krieger and Yekutieli. Significant (*p* < 0.05) differences in spirochete number relative to the *ospC* deletion strain (“*”) and between two strains relative to each other (“+”) are indicated. **(B)** Joint diameter of the mice infected with 10^4^ of *B*. *burgdorferi* strain B31-A3/pBSV2G (“B31-A3/Vector”), *ospC* deletion strain B31-A3Δ*ospC*/pBSV2G (“Vector”), or the deletion strain bearing a plasmid encoding either OspC_B31-ECM_- or OspC_B31 #2_ was measured by caliper on day 21 post infection. Data shown are the median of the diameter of ankle joints ± the range of these values in five mice per group. Statistical significance was determined using ANOVA with the Kruskal-Wallis test followed by the two-stage step-up method of Benjamini, Krieger and Yekutieli. Significant (*p* < 0.05) differences in the average diameter of ankle joint relative to the *ospC* deletion strain (“*”) and between two strains relative to each other (“+”) are indicated.
(TIF)

S5 Fig *B*. *burgdorferi* producing OspC_PBr_ triggers an adaptive immune response indistinguishable from a strain producing OspC_B31_ or OspC_N40-D10/E9_, and *B*. *burgdorferi* producing OspCB31-ECM- triggers a response indistinguishable from an *ospC*-deficient strain. Serum titers of IgG (top panel) and IgM (bottom panel) of C3H/HeN mice infected with 10^4^ *B*. *burgdorferi* strain B31-A3/pBSV2G (“B31-A3/Vector”), *ospC* deletion strain B31-A3Δ*ospC*/pBSV2G (“Vector”), or the *ospC* deletion strain bearing a plasmid encoding the indicated OspC variants sacrificed at 21 days post infection. Shown are the geometric mean of antibody titers ± 95% confidence interval of 10 mice per group. Statistical significance was determined using ANOVA with the Kruskal-Wallis test followed by the two-stage step-up method of Benjamini, Krieger and Yekutieli. Significant (*p* < 0.05) differences in the antibody titers relative to the *ospC* deletion strain (“*”) and between two strains relative to each other (“+”) are indicated.
(TIF)

S6 Fig Differences in tissue colonization promoted by distinct OspC variants are not affected by an adaptive immune response. C3H/HeN-SCID mice were intradermally inoculated with 1x10^4^ WT *B*. *burgdorferi* strain B31-A3 carrying the empty vector ("B31-A3/Vector"), B31-A3Δ*ospC* carrying the empty vector ("Vector"), B31-A3Δ*ospC* exogenously producing OspC from *B*. *burgdorferi* strain B31-A3 ("B31"), N40 clone D10/E9 ("N40-D10/E9"), or *B*. *garinii* strain PBr ("PBr") under control of the *ospC* promoter from B31-A3. Tissues were harvested at 21 days post infection, and bacterial genomes were quantified in each tissue by qPCR. Shown are the geometric mean of bacterial loads ± 95% confidence interval of 10 mice per group. Statistical significance was determined using ANOVA with the Kruskal-Wallis test followed by the two-stage step-up method of Benjamini, Krieger and Yekutieli. Significant (*p* < 0.05) differences in spirochete number relative to the *ospC* deletion strain (“*”) and between two strains relative to each other (“+”) are indicated.
(TIF)

S1 Table Bacterial strains. (PDF)

S2 Table Oligonucleotides. (PDF)

S3 Table pBSV2:chromosome ratio during in vitro culture. (PDF)

S4 Table OspC-encoding plasmids are retained at 21 days post-infection in Experiment 1. (PDF)

S5 Table OspC-encoding plasmids are retained at 21 days post-infection in Experiment 2. (PDF)

S1 Text Supplmentary materials and methods. (PDF)
