# Supplementary figures and images for: Strain-specific joint invasion and colonization by Lyme disease spirochetes is promoted by outer surface protein C
Source: PLoS Pathog. 2020 May 15;16(5):e1008516. doi: 10.1371/journal.ppat.1008516 (PMC7255614; doi:10.1371/journal.ppat.1008516)

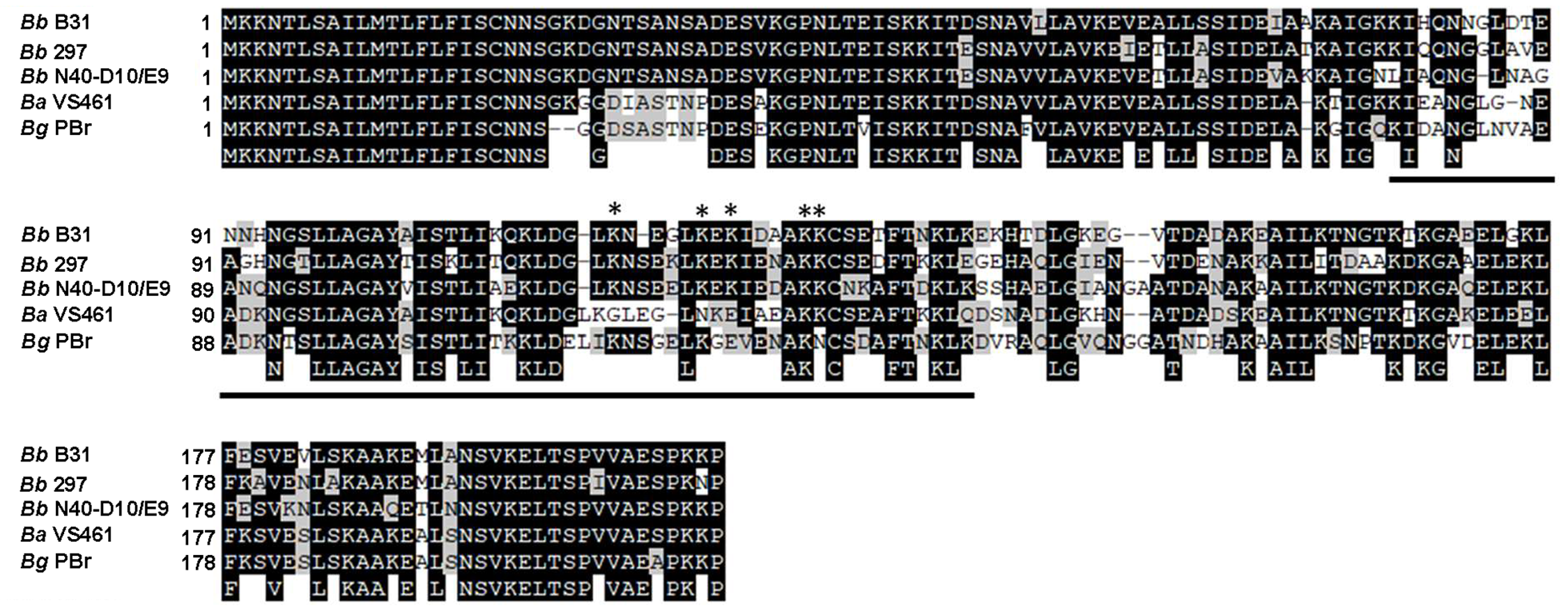

Supplement: S1 Fig — ClustalW alignment performed on protein sequences of OspC from B. burgdorferi (Bb) strain B31 (“Bb B31”), 297 (“Bb 297”), and N40-D10/E9 (“Bb N40-D10/E9”), B. afzelii strain VS461 (“Ba VS461”), and B. garinii strain PBr (“Bg PBr”) using BioEditor Sequence Alignment Editor [90]. At each position, conserved amino acids are highlighted in black and similar amino acids are highlighted in grey. Asterisks indicate OspCB31 lysines that were mutated to methionines to abrogate ECM binding, resulting in OspCB31-ECM-. The fragment of OspC from N40-D10/E9 selected for binding to joint and heart by phage display [49] is underlined. (TIF) [file ppat.1008516.s001.tif]

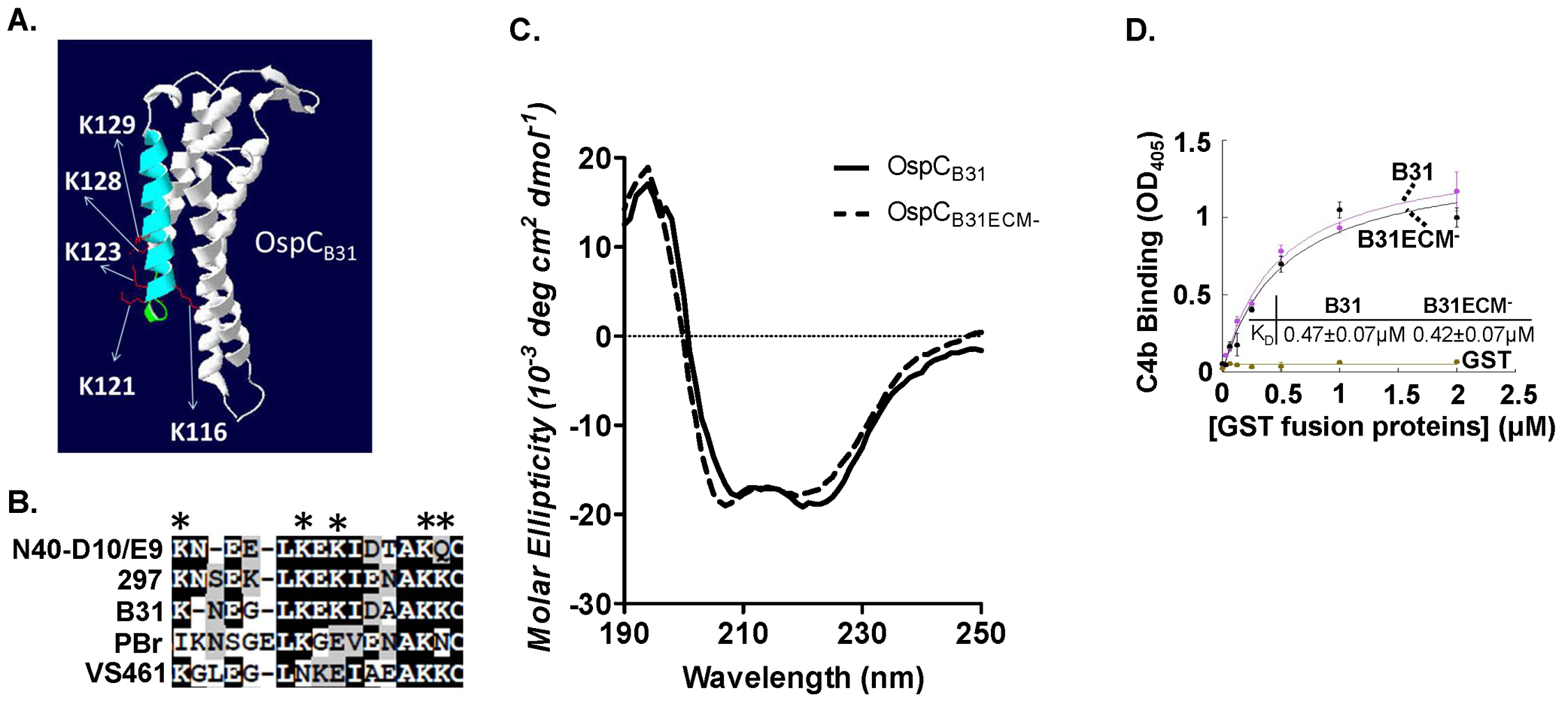

Supplement: S2 Fig — (A) The location of the quintuple basic residues (K116, K121, K123, K128, K129) mutated in OspCECM- mapped onto the crystal structure of OspCB31 [91, 92]. Helix 3 and loop 4 are highlighted in blue and green, respectively. (B) Amino acid sequence alignment of helix 3 and loop 4 of different ospC alleles. “*” indicates residues mutated in OspCB31-ECM-. (C) Far-UV CD analysis of OspCB31 and OspCB31-ECM-. The molar ellipticity, Φ, was measured from 190 to 250 nm for 10 μM of each protein in Tris buffer (pH7.5). (D) The indicated concentrations of GST, GST-OspCB31, or GST-OspCB31-ECM- were added to quadruplicate wells coated with human C4b, and binding (± standard deviation) was measured by ELISA (see Experimental Procedures). Shown is a representative of three independent experiments. The KD values obtained from the average of three independent experiments were calculated and shown in the inset. (TIF) [file ppat.1008516.s002.tif]

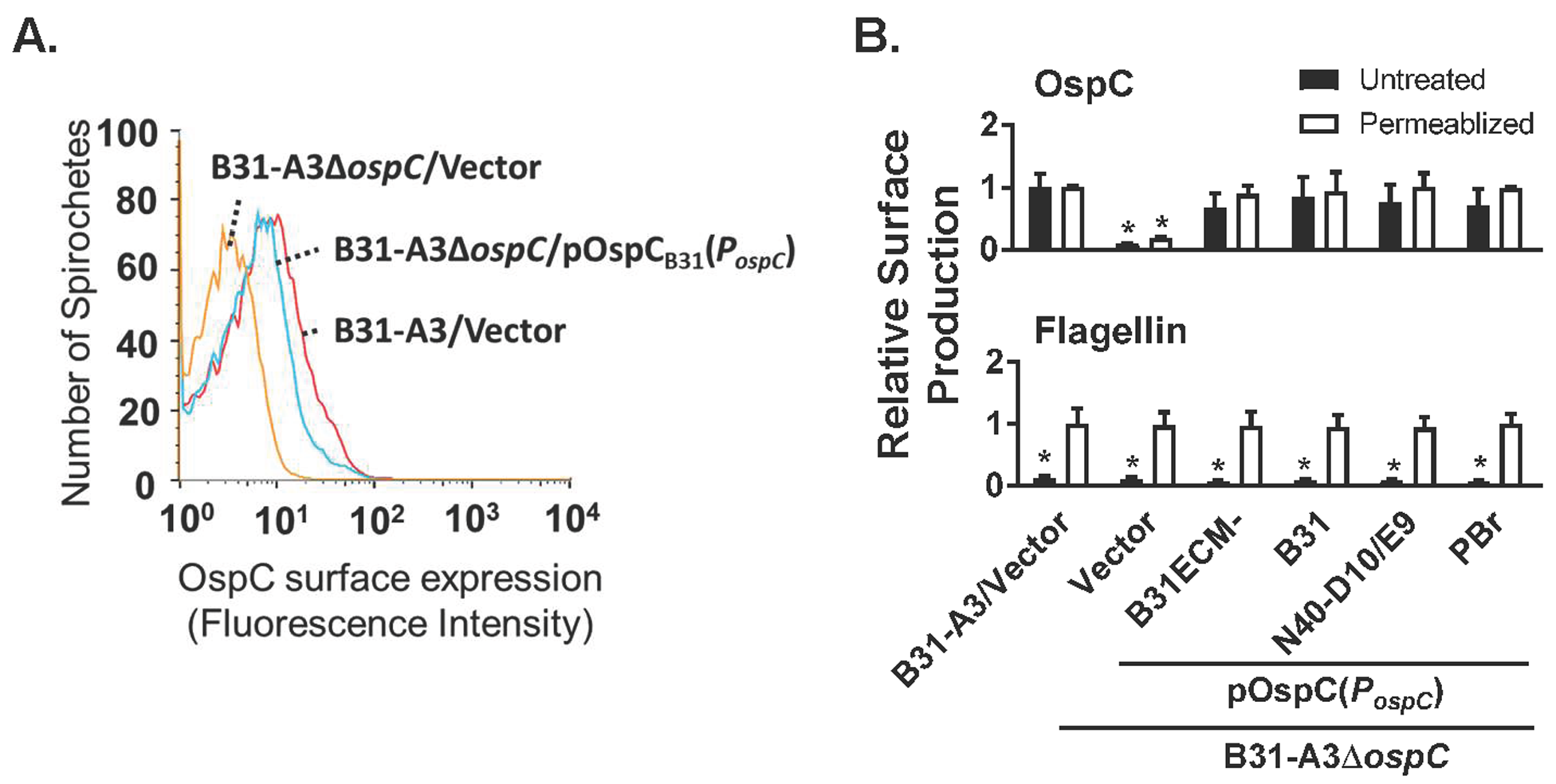

Supplement: S3 Fig — (A) Flow cytometry analysis of OspC localized to the surface of parental strain B. burgdorferi B31-A3/pBSV2G (“B31-A3/Vector”; red), ospC deletion strain B31-A3ΔospC/pBSV2G (“B31-A3ΔospC/Vector”; orange), and the ospC deletion strain bearing a plasmid encoding OspCB31 (“B31-A3ΔospC/pOspCB31”; blue). (B) OspC (top) or flagellin (bottom) on the surface of the indicated untreated (solid bars) or methanol-permeabilized (open bars) strains was quantitated by flow cytometry after staining with anti-OspC or anti-flagellin, respectively (see Materials and Methods). Values shown are relative to the production levels of OspC or flagellin on the surface of permeabilized B. burgdorferi strain B31-A3 harboring the empty vector. Each bar represents the mean of four independent determinations ± SEM. (*): indicates that surface production of the indicated proteins was significantly lower (* = P < 0.05, ANOVA with the Kruskal-Wallis test followed by the two-stage step-up method of Benjamini, Krieger and Yekutieli) than the detected production of OspC or Flagellin by B. burgdorferi strain B31-A3 harboring the vector. (TIF) [file ppat.1008516.s003.tif]

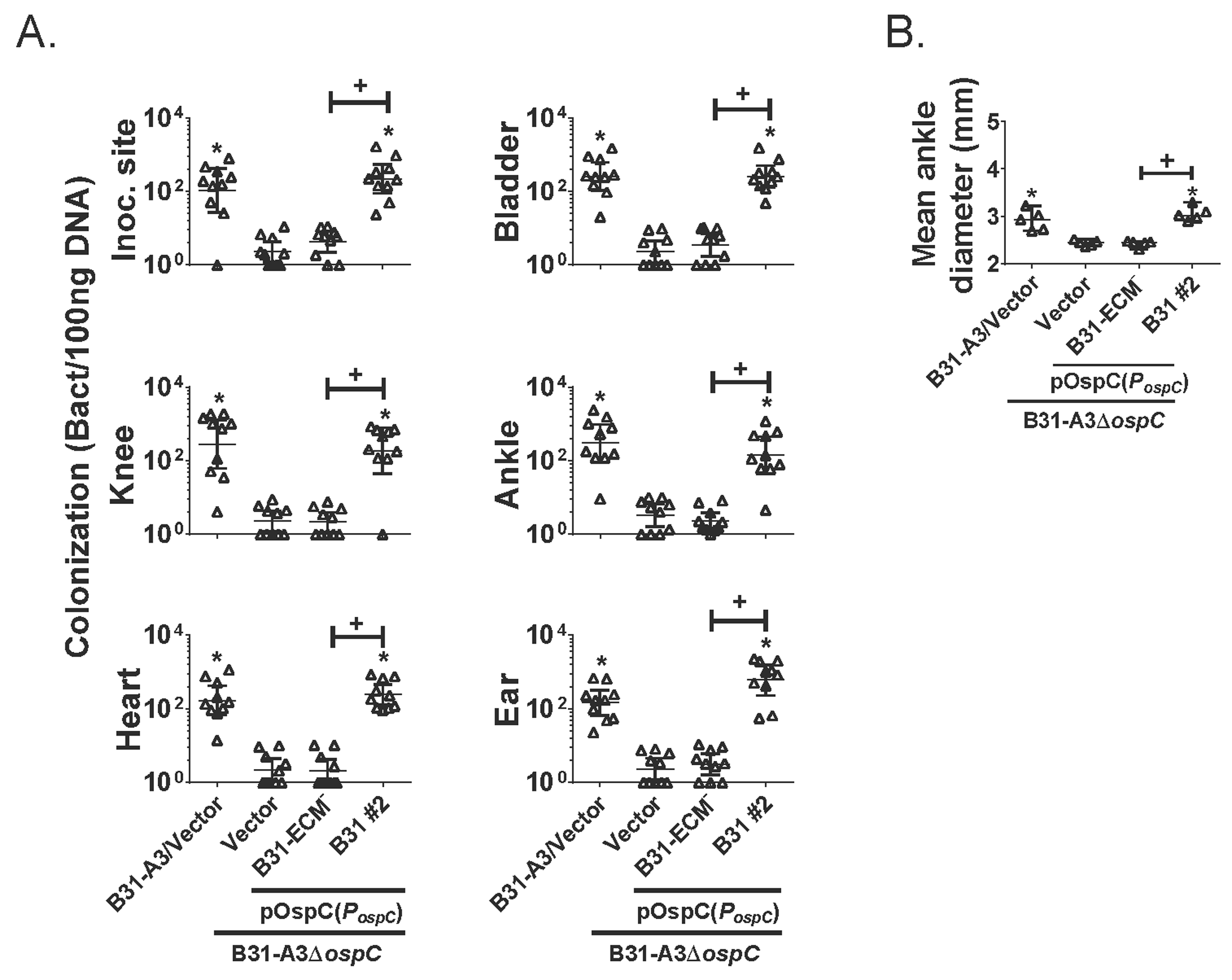

Supplement: S4 Fig — (A) C3H/HeN mice infected with 104 B. burgdorferi strain B31-A3/pBSV2G (“B31-A3/Vector”), ospC deletion strain B31-A3ΔospC/pBSV2G (“Vector”), or the deletion strain bearing a plasmid encoding either OspCB31-ECM- or OspCB31 #2, which differ only in the ospC coding sequence, were sacrificed at 21 days post infection. The bacterial loads at the inoculation site, ear, bladder, heart, knee, and ankle joint were determined by qPCR. Data shown are the geometric mean of bacterial loads ± 95% confidence interval of 10 mice per group. Statistical significance was determined using ANOVA with the Kruskal-Wallis test followed by the two-stage step-up method of Benjamini, Krieger and Yekutieli. Significant (p < 0.05) differences in spirochete number relative to the ospC deletion strain (“*”) and between two strains relative to each other (“+”) are indicated. (B) Joint diameter of the mice infected with 104 of B. burgdorferi strain B31-A3/pBSV2G (“B31-A3/Vector”), ospC deletion strain B31-A3ΔospC/pBSV2G (“Vector”), or the deletion strain bearing a plasmid encoding either OspCB31-ECM- or OspCB31 #2 was measured by caliper on day 21 post infection. Data shown are the median of the diameter of ankle joints ± the range of these values in five mice per group. Statistical significance was determined using ANOVA with the Kruskal-Wallis test followed by the two-stage step-up method of Benjamini, Krieger and Yekutieli. Significant (p < 0.05) differences in the average diameter of ankle joint relative to the ospC deletion strain (“*”) and between two strains relative to each other (“+”) are indicated. (TIF) [file ppat.1008516.s004.tif]

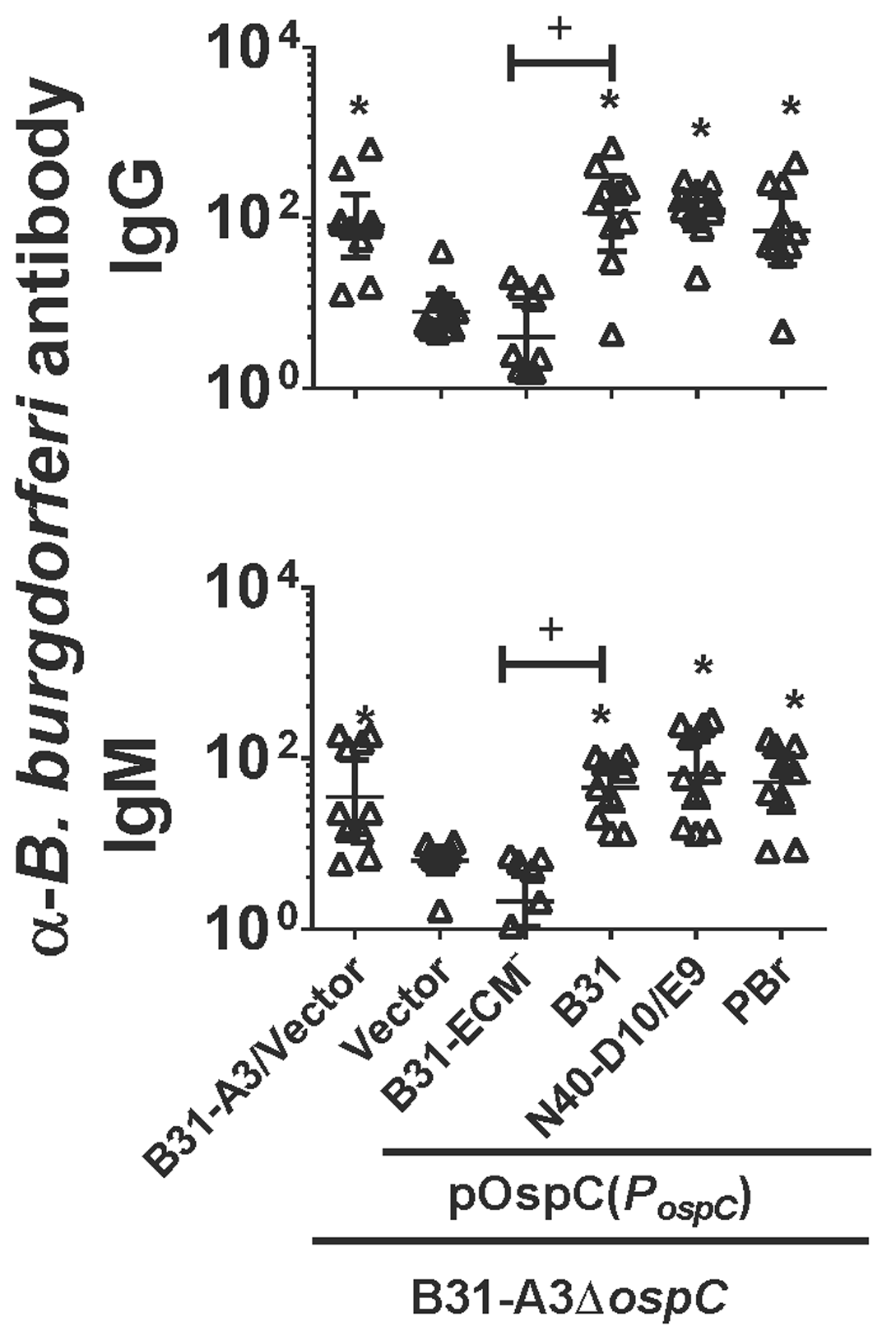

Supplement: S5 Fig — Serum titers of IgG (top panel) and IgM (bottom panel) of C3H/HeN mice infected with 104 B. burgdorferi strain B31-A3/pBSV2G (“B31-A3/Vector”), ospC deletion strain B31-A3ΔospC/pBSV2G (“Vector”), or the ospC deletion strain bearing a plasmid encoding the indicated OspC variants sacrificed at 21 days post infection. Shown are the geometric mean of antibody titers ± 95% confidence interval of 10 mice per group. Statistical significance was determined using ANOVA with the Kruskal-Wallis test followed by the two-stage step-up method of Benjamini, Krieger and Yekutieli. Significant (p < 0.05) differences in the antibody titers relative to the ospC deletion strain (“*”) and between two strains relative to each other (“+”) are indicated. (TIF) [file ppat.1008516.s005.tif]

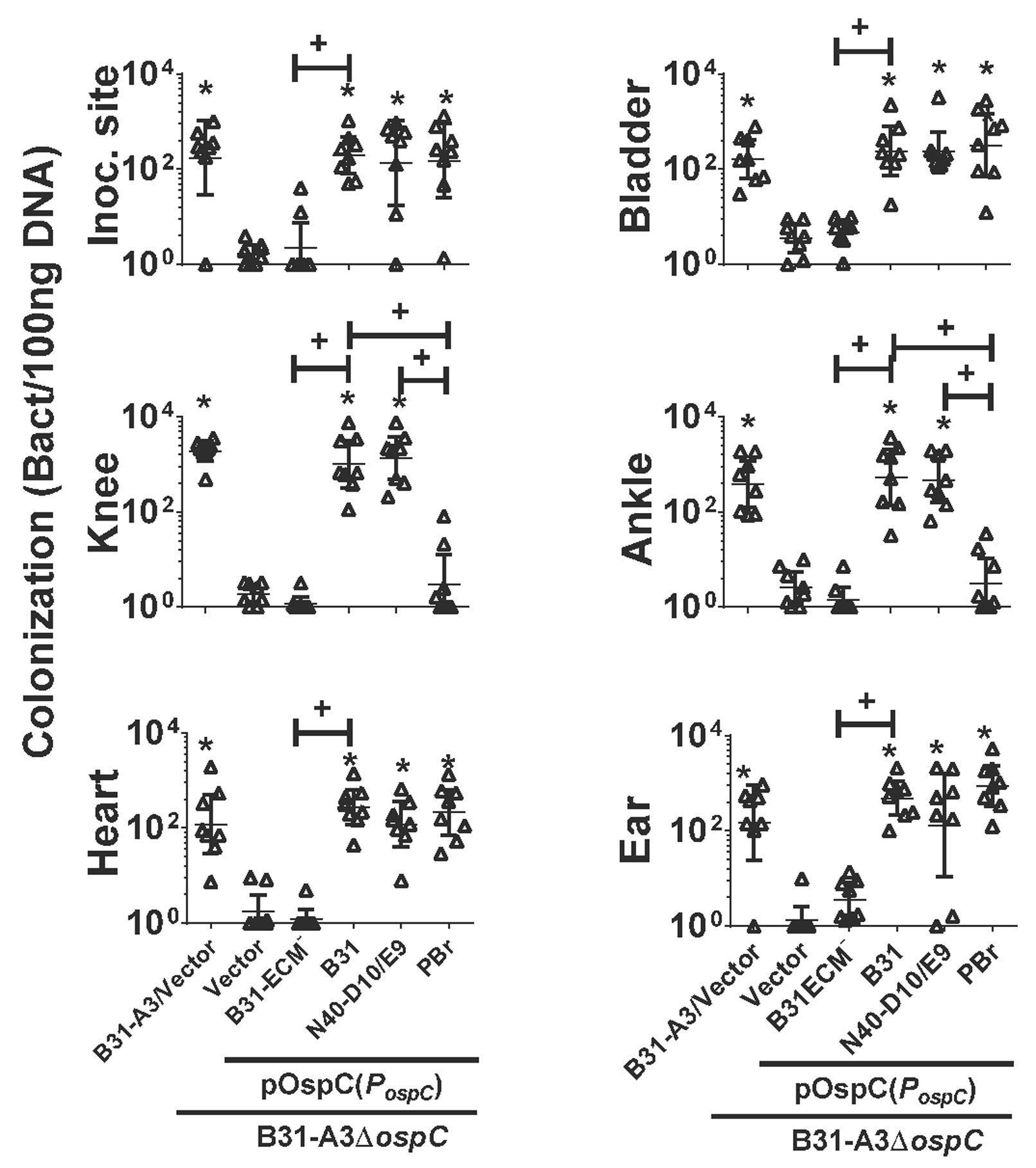

Supplement: S6 Fig — C3H/HeN-SCID mice were intradermally inoculated with 1x10^4^ WT B. burgdorferi strain B31-A3 carrying the empty vector ("B31-A3/Vector"), B31-A3ΔospC carrying the empty vector ("Vector"), B31-A3ΔospC exogenously producing OspC from B. burgdorferi strain B31-A3 ("B31"), N40 clone D10/E9 ("N40-D10/E9"), or B. garinii strain PBr ("PBr") under control of the ospC promoter from B31-A3. Tissues were harvested at 21 days post infection, and bacterial genomes were quantified in each tissue by qPCR. Shown are the geometric mean of bacterial loads ± 95% confidence interval of 10 mice per group. Statistical significance was determined using ANOVA with the Kruskal-Wallis test followed by the two-stage step-up method of Benjamini, Krieger and Yekutieli. Significant (p < 0.05) differences in spirochete number relative to the ospC deletion strain (“*”) and between two strains relative to each other (“+”) are indicated. (TIF) [file ppat.1008516.s006.tif]
