# Supplementary material for: Strain-specific joint invasion and colonization by Lyme disease spirochetes is promoted by outer surface protein C
Source: PLoS Pathog. 2020 May 15;16(5):e1008516. doi: 10.1371/journal.ppat.1008516 (PMC7255614; doi:10.1371/journal.ppat.1008516)
Supplement: S1 Table — (PDF) [file ppat.1008516.s007.pdf]

S1 Table. Bacterial strains.

**Strains**	**Description**	***Borrelia* plasmid Content***	**OspC Type**	**Reference**
B. burgdorferi sensu lato strains				
B. burgdorferi strain B31-A3	Infectious low passage clone of the tick isolate, B31	missing cp9	A	[1]
B. burgdorferi strain N40 D10/E9	Infectious low passage clone, D10/E9, of the tick isolate, N40	N.A.#	M	[2]
B. garinii strain PBr	Infectious low passage clone from human CSF	N.A.#	B	[2]
B31-A3Δ*ospC* (strain designation GCB3022)	B31-A3 deficient in OspC expression (OspCK1)	missing cp9	N/A	[3]
B31-A3/pBSV2G	*Bb* strain B31-A3 carrying the pBSV2G empty vector	missing cp9	A	[3]
B31-A3Δ*ospC*/pOspC$_{B31}$(P_{ospC})	B31-A3 Δ*ospC*::*kan* complemented with *ospC* from *B. burgdorferi* strain B31 under the control of *ospC* promoter from *B. burgdorferi* strain B31 (nucleotides −200 to −1, with 0 = **A**TG start of *ospC* coding sequence).	missing cp9	A	[3]
B31-A3Δ*ospC*/pOspC$_{B31}$ #2(P_{ospC}) (strain designation YLWB001)	B31-A3 Δ *ospC*::*kan* complemented with *ospC* from *B. burgdorferi* strain B31 under the control of *ospC* promoter from *B. burgdorferi* strain B31 (nucleotides −184 to −9, with 0 = **A**TG start of *ospC* coding sequence).	missing cp9	A	This study
B31-A3Δ*ospC*/pOspC$_{N40\text{-}D10/E9}$ (P_{ospC}) (strain designation YLB97)	B31-A3 Δ *ospC*::*kan* complemented with *ospC* from *B. burgdorferi* strain N40-D10/E9 under the control of *ospC* promoter from *B. burgdorferi* strain B31	missing cp9	M	[4]

	(nucleotides −184 to −9, with 0 = **A**TG start of *ospC* coding sequence).			
B31-A3Δ*ospC*/pOspC$_{PBr}$(P_{ospC}) (strain designation YLB98)	B31-A3 Δ *ospC*::*kan* complemented with *ospC* from *B. garinii* strain PBr under the control of *ospC* promoter from *B. burgdorferi* strain B31 (nucleotides −184 to −9, with 0 = **A**TG start of *ospC* coding sequence).	missing cp9	B	[4]
B31-A3Δ*ospC*/pOspC$_{B31\text{-}ECM^{-}}$(P_{ospC}) (strain designation YLB99)	B31-A3 Δ *ospC*::*kan* complemented with *ospC* from *B. burgdorferi* strain B31 with Lysine-116, -121, -123, -128, and -129 replaced by methionine residues under the control of *ospC* promoter from *B. burgdorferi* strain B31 (nucleotides −184 to −9, with 0 = **A**TG start of *ospC* coding sequence).	missing cp9	A	This study
B31-A3Δ*ospC*/pBSV2G (strain designation YLB107)	*Bb* strain B31-A3Δ*ospC* carrying the pBSV2G empty vector	missing cp9	N/A	[3]
B31-A3Δ*ospC*/pOspCB31(P_{flaB})	*Bb* strain B31-A3Δ*ospC* expressing OspC from *Bb* strain B31-A3 from the *flaB* promoter	missing cp9	A	[5]
B31-A3Δ*ospC*/pOspC$_{N40\text{-}D10/E9}$(P_{flaB})	*Bb* strain B31-A3Δ*ospC* expressing OspC from *Bb* strain N40 D10/E9, containing a single amino acid mutation at N53 to a serine, from the *flaB* promoter	missing cp9	M	[4]
B31-A3Δ*ospC*/pOspC$_{PBr}$(P_{flaB})	*Bb* strain B31-A3Δ*ospC* expressing OspC from *Bg* strain PBr from the *flaB* promoter	missing cp9	B	[4]
B31-A3/pTM61 (strain designation GCB847)	*Bb* strain B31-A3 carrying the pTM61 vector, the pBSV2G vector (gentamicin resistant) encoding a green fluorescence protein driven by *flgB* promoter.	missing cp9	N/A	[6] [7]
B31-A3Δ*ospC*/pTM61spc (strain designation GCB3007)	*Bb* strain B31-A3Δ*ospC* carrying the pTM61spc vector (streptomycin resistant), the pTM61 vector with a gentamicin resistance gene replaced by a streptomycin resistance gene.	missing cp9	N/A	This study

B31-A3Δ*ospC*/pTM61spc-$OspC_{PBr}$(P_{flaB}) (strain designation GCB3226)	*Bb* strain B31-A3Δ*ospC* carrying the pTM61spc vector encoding *ospC* from *Bg* strain PBr driven by the *flaB* promoter	missing cp9	B	This study
B31-A3Δ*ospC*/pTM61-$OspC_{B31}$(P_{ospC}) (strain designation GCB4458)	*Bb* strain B31-A3Δ*ospC* carrying the pTM61 vector encoding o*spC* from *Bb* strain B31-A3 from the P_{ospC} promoter	missing lp28-4, lp56, cp9	A	This study
B31-A3Δ*ospC*/pTM61-$OspC_{B31\text{-}ECM}^{-}$($P_{ospC}$) (strain designation GCB4452)	*Bb* strain B31-A3Δ*ospC* carrying the pTM61 vector encoding $ospC_{B31\text{-}ECM}^{-}$ mutant (ospC from *Bb* strain B31-A3 with lysines 116, 121, 123, 128, 129 mutated to methionine) under the P_{ospC} promoter	missing cp9	A	This study
E. coli strains				
DH5α	F^- *mcr*A, Δ(*mrr-hsd*RMS-*mcr*BC), φ80*lac*ZΔM15, Δ*lac*X74, *rec*A1, *end*A1, *ara*D139Δ(*ara, leu*)7697, *gal*U, *gal*K, λ^- *rps*L, *nup*G	N/A	N/A	Invitrogen, Houston, TX
BL21	$F^–$, *ompT, hsd*SB ($rB^–$, $mB^–$), *dcm, gal*, λ(DE3)	N/A	N/A	Promega Corp., Madison, WI
BL21/pGEX4T2-$OspC_{B31}$ (strain designation YLE53)	BL21 expressing GST-tagged OspC from *B. burgdorferi* strain B31	N/A	A	[4]
BL21/pGEX4T2-$OspC_{N40\text{-}D10/E9}$ (strain designation YLE26)	BL21 expressing GST-tagged OspC from *B. burgdorferi* strain N40-D10/E9	N/A	M	[4]
BL21/pGEX4T2-$OspC_{PBr}$ (strain designation YLE57)	BL21 expressing GST-tagged OspC from *B. garinii* strain PBr	N/A	B	[4]
BL21/pGEX4T2-$OspC_{B31\text{-}ECM}^{-}$ (strain designation YLE173)	BL21 expressing GST-tagged OspC from *B. burgdorferi* strain B31 with Lysine-116, -121, -123, -128, and -129 replaced by methionine residues	N/A	A	This study
GCE3815	DH5α carrying pMC114	N/A	N/A	This study

GCE3817	DH5α carrying pMC115	N/A	N/A	This study

*****=Genomic plasmid content as determined by PCR using primers developed using *B. burgdorferi* strain B31 genomic DNA sequences.
[#]= Not applicable. The appropriate genomic plasmid-specific primer sets have not been designed for these strains and thus these strains' plasmid profiles could not be determined using the primers designed for B31 strains. However, these strains were cultivated less than 10 passages to avoid the potential plasmid missing.
cp= circular plasmid
lp= linear plasmid

Reference

1. Elias AF, Stewart PE, Grimm D, Caimano MJ, Eggers CH, Tilly K, et al. Clonal polymorphism of *Borrelia burgdorferi* strain B31 MI: implications for mutagenesis in an infectious strain background. Infect Immun. 2002;70(4):2139-50.
2. Coburn J, Barthold SW, Leong JM. Diverse Lyme disease spirochetes bind integrin alpha IIb beta 3 on human platelets. Infect Immun. 1994;62(12):5559-67.
3. Tilly K, Krum JG, Bestor A, Jewett MW, Grimm D, Bueschel D, et al. *Borrelia burgdorferi* OspC protein required exclusively in a crucial early stage of mammalian infection. Infect Immun. 2006;74(6):3554-64.
4. Caine JA, Lin YP, Kessler JR, Sato H, Leong JM, Coburn J. *Borrelia burgdorferi* outer surface protein C (OspC) binds complement component C4b and confers bloodstream survival. Cell Microbiol. 2017. doi: 10.1111/cmi.12786. PubMed PMID: 28873507.
5. Caine JA, Coburn J. A short-term *Borrelia burgdorferi* infection model identifies tissue tropisms and bloodstream survival conferred by adhesion proteins. Infect Immun. 2015;83(8):3184-94.
6. Kumar D, Ristow LC, Shi M, Mukherjee P, Caine JA, Lee WY, et al. Intravital Imaging of Vascular Transmigration by the Lyme Spirochete: Requirement for the Integrin Binding Residues of the *B. burgdorferi* P66 Protein. PLoS Pathog. 2015;11(12):e1005333.
7. Moriarty TJ, Norman MU, Colarusso P, Bankhead T, Kubes P, Chaconas G. Real-time high resolution 3D imaging of the lyme disease spirochete adhering to and escaping from the vasculature of a living host. PLoS Pathog. 2008;4(6):e1000090.
