# Supplementary material for: Strain-specific joint invasion and colonization by Lyme disease spirochetes is promoted by outer surface protein C
Source: PLoS Pathog. 2020 May 15;16(5):e1008516. doi: 10.1371/journal.ppat.1008516 (PMC7255614; doi:10.1371/journal.ppat.1008516)
Supplement: S2 Table — (PDF) [file ppat.1008516.s008.pdf]

S2 Table. Oligonucleotides#

Primer/Vector	Sequence (5' to 3') *	Amplified DNA fragment	Reference
$OspC_{B31}$pfp/pGEX4T2	cgGGATCCaaagatgggaatacatct	*$OspC_{B31}p$*	[1]
$OspC_{B31}$prp/pGEX4T2	cgGTCGACttaaggttttttggact		[1]
$OspC_{N40-D10/E9}$/pGEX4T2	cgGGATCCaaagatgggaatacatct	*$ospC_{N40-D10/E9}p$*	[1]
$OspC_{N40-D10/E9}$/pGEX4T2	cgGTCGACttaaggttttttggact		[1]
$OspC_{PBr}$pfp/pGEX4T2	cgGGATCCgattctgcatctactaat	*$ospC_{PBr}p$*	[1]
$OspC_{PBr}$prp/pGEX4T2	cgGTCGACttaaggttttttggagc		[1]
$OspC_{B31-ECM^-}$pfp/pGEX4T2	cgGGATCCagtttaacaggaaaagct	*$ospC_{B31-ECM^-}p$*	This study
$OspC_{B31-ECM^-}$prp/pGEX4T2	cgGTCGACttccacttttgctttcat		This study
pospCfp/pBSV2G	cgGCATGCggcaaactggaaacaagt	*pospC*	[2]
pospCrp/pBSV2G	cgGTCGACtttttcctccttctatta		[2]
$OspC_{N40-D10/E9}$fp/pBSV2G	cgGTCGACatgaaaaagaatacatta	*$ospC_{N40-D10/E9}$*	[1]
$OspC_{N40-D10/E9}$rp/pBSV2G	cgGGATCCttaaggttttttggact		[1]
$OspC_{PBr}$fp/pBSV2G	cgGTCGACatgaaaaagaatacatta	*$ospC_{PBr}$*	[1]
$OspC_{PBr}$rp/pBSV2G	cgGGATCCttaaggttttttggagc		[1]
$OspC_{B31}$fp/pTM61 (B2949)	tttCCTGCAGGttaattttagcatatttggctt	*$ospC_{B31}$ and $ospC_{B31-ECM^-}$*	This study
$OspC_{B31}$rp/pTM61 (B2956)	tttCCTAGGttaaggttttttggactttc		This study
$OspC_{B31-ECM^-}$1	tttcagaacacatcatagccgcatcaatcatttc cattaatccttcattcatcaatccatctaatttttgtt ttattagggttga	*$ospC_{B31-ECM^-}$*	This study
$OspC_{B31-ECM^-}$2	agatggattgatgaatgaaggattaatggaaa tgattgatgcggctatgatgtgttctgaaacattt actaataaattaaaagaa		This study
$OspC_{B31-ECM^-}$3	Aatttttgttttattagggttga		This study
$OspC_{B31-ECM^-}$4	Catttactaataaattaaaagaa		This study
BBRecAfp	gtggatctattgtattagatgaggctctcg	*BbRecA*	[3]
BBRecArp	gccaaagttctgcaacattaacacctaaag		[3]

Genfp	atgttacgcagcagcaac	gentamycin resistance cassette	[4]
Genrp	ttaggtggcggtacttgg		[4]
ColE1fp	ctacatacctcgctctgctaatc	pBSV2G origin of replication	[5]
ColE1rp	cgaaacccgacaggactataaa		[5]
oMCS3	tcatgaggcgcgccagagctcgagagagctagcagagggccctcatgaa	MCS inserted into pTM61spc (169) to generate pTM61spc-MCS	This study
oMCS4	tcatgagggccctctgctagctctctcgagctctggcgcgcctcatgaa		This study
PFlaB.Fwd	gagagctagctgtctgtcgcctcttgtgg	Amplification of the flagellar promoter from B. burgdorferi strain B31-A3 for cloning into pGEM ™-T Easy (Promega corp., Madison, WI)	This study
PFlaB.Rev	gagagggccctatatcattcctccatgataaaattt		This study

* The restriction sites used are shown as capital letters.

# The primers for plasmid profiling have been described [6, 7]. These primers are thus not included.

References

1. Caine JA, Lin YP, Kessler JR, Sato H, Leong JM, Coburn J. *Borrelia burgdorferi* outer surface protein C (OspC) binds complement component C4b and confers bloodstream survival. Cell Microbiol. 2017. doi: 10.1111/cmi.12786.

2. Fischer JR, LeBlanc KT, Leong JM. Fibronectin binding protein BBK32 of the Lyme disease spirochete promotes bacterial attachment to glycosaminoglycans. Infect Immun. 2006;74(1):435-41.

3. Liveris D, Wang G, Girao G, Byrne DW, Nowakowski J, McKenna D, et al. Quantitative detection of *Borrelia burgdorferi* in 2-millimeter skin samples of erythema migrans lesions: correlation of results with clinical and laboratory findings. J Clin Microbiol. 2002;40(4):1249-53.

4. Lin YP, Benoit V, Yang X, Martinez-Herranz R, Pal U, Leong JM. Strain-specific variation of the decorin-binding adhesin DbpA influences the tissue tropism of the lyme disease spirochete. PLoS Pathog. 2014;10(7):e1004238.

5. Marcinkiewicz AL, Dupuis AP, 2nd, Zamba-Campero M, Nowak N, Kraiczy P, Ram S, et al. Blood treatment of Lyme borreliae demonstrates the mechanism of CspZ-mediated complement evasion to promote systemic infection in vertebrate hosts. Cell Microbiol. 2019;21(2):e12998.

6. Purser JE, Norris SJ. Correlation between plasmid content and infectivity in *Borrelia burgdorferi*. Proc Natl Acad Sci U S A. 2000;97(25):13865-70.

7. Bunikis I, Kutschan-Bunikis S, Bonde M, Bergstrom S. Multiplex PCR as a tool for validating plasmid content of *Borrelia burgdorferi*. J Microbiol Methods. 2011;86(2):243-7.
