# Supplementary material for: Strain-specific joint invasion and colonization by Lyme disease spirochetes is promoted by outer surface protein C
Source: PLoS Pathog. 2020 May 15;16(5):e1008516. doi: 10.1371/journal.ppat.1008516 (PMC7255614; doi:10.1371/journal.ppat.1008516)
Supplement: S3 Table — (PDF) [file ppat.1008516.s009.pdf]

S3 Table. pBSV2:chromosome ratio during *in vitro* culture.

Strain		Bacteria/5pg DNA[a]		
		In vitro		
		Plasmid[b]	Chrom[c]	Plasmid /Chrom[d]
B31-A3/Vector		722.1 ±2.8	742.9 ±2.8	**0.97**
B31-A3Δ*ospC*	Vector	804.7 ±2.9	813.6 ±2.9	**0.98**
	pOspC$_{B31\ \#2}$	669.0 ±2.8	743.9 ±2.8	**0.89**
	pOspC$_{B31\text{-}ECM^-}$	701.7 ±2.8	736.4 ±2.8	**0.95**

[a] Experiment displayed in Figure S4.
[b] Spirochetal burden determined by qPCR; shown are geometric mean ± geometric standard deviation from 10 mice.
[c] Spirochetal burden determined using *colE1* primers.
[d] Spirochetal burden determined using *recA* primers.
[e] Ratio of burden determined using *colE1* primers to burden determined using *recA* primers.
[f] NA, not applicable because the burdens obtained using either *recA* primers, *colE1* primers, or both were below the detection limit of 10 bacterial copies per 100ng DNA.
