# Supplementary material for: Strain-specific joint invasion and colonization by Lyme disease spirochetes is promoted by outer surface protein C
Source: PLoS Pathog. 2020 May 15;16(5):e1008516. doi: 10.1371/journal.ppat.1008516 (PMC7255614; doi:10.1371/journal.ppat.1008516)
Supplement: S4 Table — (PDF) [file ppat.1008516.s010.pdf]

S4 Table. OspC-encoding plasmids are retained at 21 days post-infection in Experiment 1[a].

Strain		Spirochetal burden (Bacteria/100ng DNA)[b]								
		Inoculation Site			Tibiotarsus			Heart		
		Plasmid[c]	Chrom[d]	Plasmid /Chrom[e]	Plasmid	Chrom	Plasmid /Chrom	Plasmid	Chrom	Plasmid /Chrom
B31-A3/Vector		171.6 ±5.1	177.6 ±5.1	**0.96**	658.7 ±8.5	794.9 ±11.6	**0.82**	95.5 ±2.2	114.9± 7.7	**0.83**
B31-A3Δ*ospC*	Vector	1.3 ±1.5	2.3 ±2.4	**N.A.**[f]	3.2 ±2.1	3.7 ±2.6	**N.A.**	3.5 ±2.1	2.0 ±1.9	**N.A.**
	pOspC$_{B31}$	69.2 ±4.8	85.3 ±12.7	**0.81**	519.4 ±8.0	648.2 ±4.0	**0.80**	112.2 ±4.8	135.5± 2.3	**0.82**
	pOspC$_{N40-D10/E9}$	60.4 ±5.1	61.2 ±7.6	**0.98**	185.6 ±6.5	173.7 ±7.1	**1.06**	208.1 ±5.6	256.7± 2.0	**0.81**
	pOspC$_{PBr}$	67.2 ±8.2	67.6 ±9.9	**0.99**	4.5 ±2.7	10.0 ±3.7	**N.A.**	63.5 ±4.5	64.4 ±6.4	**0.98**
	pOspC$_{B31-ECM^-}$	1.4 ±1.6	2.3 ±2.8	**N.A.**	1.3 ±1.9	2.5 ±2.6	**N.A.**	2.6 ±2.3	3.8 ±2.9	**N.A.**

[a] Experiment displayed in Figure 5.
[b] Spirochetal burden determined by qPCR; shown are geometric mean ± geometric standard deviation from 10 mice.
[c] Spirochetal burden determined using *colE1* primers.
[d] Spirochetal burden determined using *recA* primers.
[e] Ratio of burden determined using *colE1* primers to burden determined using *recA* primers.
[f] NA, not applicable because the burdens obtained using either *recA* primers, *colE1* primers, or both were below the detection limit of 10 bacterial copies per 100ng DNA.
