# Supplementary material for: Strain-specific joint invasion and colonization by Lyme disease spirochetes is promoted by outer surface protein C
Source: PLoS Pathog. 2020 May 15;16(5):e1008516. doi: 10.1371/journal.ppat.1008516 (PMC7255614; doi:10.1371/journal.ppat.1008516)
Supplement: S5 Table — (PDF) [file ppat.1008516.s011.pdf]

S5 Table. OspC-encoding plasmids are retained at 21 days post-infection in Experiment 2[a].

Strain		Spirochetal burden								
		Bacteria/100ng DNA[b]								
		Inoculation Site			Tibiotarsus			Heart		
		Plasmid[c]	Chrom[d]	Plasmid /Chrom[e]	Plasmid	Chrom	Plasmid /Chrom	Plasmid	Chrom	Plasmid /Chrom
B31-A3/Vector		93.2 ±4.9	106.7 ±6.8	**0.87**	544.0 ±5.4	644.9 ±5.6	**0.84**	141.0 ±8.5	170.7± 3.6	**0.82**
B31-A3Δ*ospC*	Vector	1.9 ±2.3	2.2 ±2.4	**N.A.**[f]	2.9 ±2.6	3.3 ±2.7	**N.A.**	1.9 ±2.1	2.2 ±2.6	**N.A.**
	pOspC$_{B31}$	180.4 ±4.2	220.7 ±3.5	**0.81**	488.6 ±3.8	554.1 ±6.5	**0.89**	243.3 ±5.4	246.4± 2.3	**0.98**
	pOspC$_{B31\text{-}ECM^-}$	1.9 ±2.6	4.2 ±2.4	**N.A.**	1.7 ±2.3	2.3 ±2.0	**N.A.**	2.6 ±2.3	2.4 ±2.2	**N.A.**

[a] Experiment displayed in Figure S4.
[b] Spirochetal burden determined by qPCR; shown are geometric mean ± geometric standard deviation from 10 mice.
[c] Spirochetal burden determined using *colE1* primers.
[d] Spirochetal burden determined using *recA* primers.
[e] Ratio of burden determined using *colE1* primers to burden determined using *recA* primers.
[f] NA, not applicable because the burdens obtained using either *recA* primers, *colE1* primers, or both were below the detection limit of 10 bacterial copies per 100ng DNA.
