# Supplementary material for: Strain-specific joint invasion and colonization by Lyme disease spirochetes is promoted by outer surface protein C
Source: PLoS Pathog. 2020 May 15;16(5):e1008516. doi: 10.1371/journal.ppat.1008516 (PMC7255614; doi:10.1371/journal.ppat.1008516)
Supplement: S1 Text — (PDF) [file ppat.1008516.s012.pdf]

SUPPLEMENTARY MATERIALS AND METHODS

Circular dichroism (CD) spectroscopy

CD analysis was performed on a Jasco 810 spectropolarimeter (Jasco Analytical Instrument, Easton, MD) under N_2. CD spectra were measured at 25°C in a 1 mm path length quartz cell. Spectra of $OspC_{B31}$ (10 μM) and $OspC_{B31\text{-}ECM}^{-}$ (10 μM) were recorded in Tris buffer, and three far-UV CD spectra were recorded from 190 to 250 nm in 1 nm increments. The background spectrum of buffer without protein was subtracted from the protein spectra, and CD spectra were initially analyzed by the Spectra Manager program. Analysis of the spectra to extrapolate secondary structures was performed by Dichroweb (http://www.cryst.bbk.ac.uk/cdweb/html/home) using the K2D and Selcon 3 analysis programs [1].

Determination of surface exposure of OspC derivatives in *B. burgdorferi*.

Surface exposure of OspC derivatives in *B. burgdorferi* was verified by flow cytometry [2] or surface proteolysis [3]. To determine surface localization of OspC using flow cytometry, 1 × 10^8 *B. burgdorferi* cells were washed three times in HBSC buffer containing DB (25mM HEPES acid, 150mM sodium chloride, 1mM $MnCl_2$, 1mM $MgCl_2$, 0.25mM $CaCl_2$, 0.1% glucose, and 0.2% BSA, pH 7.8), and then suspended into the same buffer. To permeabilize spirochetes, pellets of 1 × 10^8 *B. burgdorferi* cells obtained after centrifugation of the cultures were suspended into 100% ethanol and then incubated for 1 h, followed by washing three times with HBS buffer containing DB. The spirochetes were ultimately suspended into HBS buffer containing DB. The suspensions of both intact and permeabilized spirochetes were incubated with a mixture of equal amounts of mouse antisera raised against $OspC_{B31}$, $OspC_{N40\text{-}D10/E9}$, and $OspC_{PBr}$ [3, 4] or the monoclonal antibody against FlaB CB1 (control) as primary antibodies (1: 250). Alexa488-conjugated goat anti-mouse IgG (Invitrogen, Carlsbad, CA ; 1: 250) was applied as the secondary antibody. 300μl of formalin (0.1%) was then added to fix the bacteria and

antibodies, and the surface localization of OspC (or FlaB) was measured by flow cytometry using a Becton-Dickinson FACSCalibur (BD Bioscience, Franklin Lakes, NJ). The mean fluorescence index (MFI) of each sample was obtained from FlowJo software version 10 (Three star Inc, Ashland, OR), representing the surface production of indicated proteins. The resulting MFI of different strains was normalized to that of permeabilized *B. burgdorferi* strain B31-A3 harboring the empty vector. The results shown in S3 Fig. represent the mean of four independent determinations ± SEM. Each SEM value was no more than seven percent of its mean value.

Construction of pOspC$_{B31\ \#2}$. To ascertain the different phenotypes between pOspC$_{B31}$- and pOspC$_{B31\text{-}ECM}^{-}$ -producing strains is not due to the differences of the 5’ non-coding sequences to the *ospC* ATG start codon between those strains, we generated the *ospC*$_{B31}$-encoding plasmid carrying the same 5’ non-coding sequences as that of pOspC$_{B31\text{-}ECM}^{-}$, pOspC$_{B31\ \#2}$. The pGEM-T Easy derived plasmids encoding *ospC*$_{B31}$ were digested with SalI and BamHI and *ospC* alleles were subcloned into pBSV2G at the SalI and BamHI sites [5]. The promoter region of *ospC* from *B. burgdorferi* B31-A3, 184 bp to 9 bp upstream of the start codon of *ospC* [6], was also PCR amplified, adding HindIII and SalI sites at the 5’and 3’ ends, respectively, using primers pospCfp and pospCrp, and subcloned into the above mentioned pBSV2G encoding *ospC*$_{B31\ \#2}$ (S2 Table).

Verification of plasmid retention in spirochetes during infection. pOspC plasmids are not stably retained in an *ospC*-deficient *B. burgdorferi* at 6 weeks post infection [7]. In addition, the pOspC plasmids utilized in this study were found to harbor a deletion of nucleotide 67 in *orf2* (P. Rosa, personal communication), resulting in a frameshift mutation on this gene. *orf2* along with the upstream and downstream genes *orf1* and *orf3* promotes autonomous replication of pBSV2G-derived plasmids such as pOspC in low passage *B. burgdorferi* [8]. To test whether

the pOspC plasmids were stably maintained in these *ospC*-complemented strains at 21d.p.i., *B. burgdorferi* pBSV2G-derived plasmids in the inoculation site of skin, heart, and ankle were quantified by qPCR (95.0°C for 5min, 95.0°C for 10 sec, 49.3°C for 30 sec repeated 40 times) using primers to amplify *colE1* origin of replication of pOspC (S2 Table) [8]. The ratio of *colE1* to *recA* copies for *in vitro*-cultivated spirochetes harboring pBSV2G, pOspC$_{B31\ \#2}$, or pOspC$_{B31\text{-}ECM}^{-}$ varied between 0.89 and 0.98 (S3 Table). The ratio of *colE1* to *recA* copies for spirochetes harboring pOspC$_{B31}$, pOspC$_{B31\ \#2}$, pOspC$_{N40\text{-}D10/E9}$ or pOspC$_{PBr}$ in colonized tissues varied between 0.81 and 1.06 (S4 and S5 Tables), indicating that vast majority of spirochetes in these tissues retained the pOspC plasmids at 21 days post infection.

Antibody titer determinations

The antibody titer was determined as described [2]. Basically, recombinant GST-OspC$_{B31}$, -OspC$_{N40\text{-}D10/E9}$, and -OspC$_{PBr}$ proteins were mixed in coating buffer (0.05 M Na_2CO_3, pH 9.0) at 10 µg/ml for each protein. Nunc maxisorp flat-bottom 96-well plates were then coated with 100 µl of the OspC protein mixture overnight at 4°C. Plates were washed with wash buffer (PBS + 0.05% Tween 20) three times and blocked for 1 hr in blocking buffer (PBS + 0.05% Tween 20 + 1% BSA) then washed as above. Each tested serum sample was diluted 1:100, 1:300, and 1:900, and incubated with the OspC-coated wells at ambient temperature for one hr. After washing, the wells were incubated in secondary antibody (HRP-conjugated goat anti-mouse IgM or IgG antibodies) (Bethyl Lab, Montgomery, TX) for one hr. After washing, the developing solution (SureBlue Reserve TMB 2-Component Microwell Peroxidase Substrate system (KPL)) was added to each well. Plates were then read at OD_{650} every minute for ten minutes using a Synergy HT ELISA plate reader (BioTek, Winooski, VT). The mean of the increasing OD_{650} values per minitue was calculated to indicate the slope of increasing OD values. Among the slopes obtained from three dilutions of a particular serum sample, the maximal slope was used to represent the antibody titers of that sample. Controls included three dilutions (1:100, 1:300

and 1:900) of purified IgG or IgM (125 mg/ml; Bethyl Lab, Montgomery, TX) coated on microtiter plates, and uninfected (‘‘naive’’) serum run in parallel with sample sera. Antibody titers in serum samples were normalized by subtracting the antibody unit ‘‘background’’ of naive mice, and expressed relative to the control wells coated with purified IgG and IgM.

References

1. Lin YP, Greenwood A, Nicholson LK, Sharma Y, McDonough SP, Chang YF. Fibronectin binds to and induces conformational change in a disordered region of leptospiral immunoglobulin-like protein B. J Biol Chem. 2009;284(35):23547-57.
2. Lin YP, Benoit V, Yang X, Martinez-Herranz R, Pal U, Leong JM. Strain-specific variation of the decorin-binding adhesin DbpA influences the tissue tropism of the lyme disease spirochete. PLoS Pathog. 2014;10(7):e1004238.
3. Caine JA, Lin YP, Kessler JR, Sato H, Leong JM, Coburn J. *Borrelia burgdorferi* outer surface protein C (OspC) binds complement component C4b and confers bloodstream survival. Cell Microbiol. 2017. doi: 10.1111/cmi.12786.
4. Benoit VM, Fischer JR, Lin YP, Parveen N, Leong JM. Allelic variation of the Lyme disease spirochete adhesin DbpA influences spirochetal binding to decorin, dermatan sulfate, and mammalian cells. Infect Immun. 2011;79(9):3501-9.
5. Purser JE, Lawrenz MB, Caimano MJ, Howell JK, Radolf JD, Norris SJ. A plasmid-encoded nicotinamidase (PncA) is essential for infectivity of *Borrelia burgdorferi* in a mammalian host. Mol Microbiol. 2003;48(3):753-64.
6. Fischer JR, LeBlanc KT, Leong JM. Fibronectin binding protein BBK32 of the Lyme disease spirochete promotes bacterial attachment to glycosaminoglycans. Infect Immun. 2006;74(1):435-41.

7. Tilly K, Krum JG, Bestor A, Jewett MW, Grimm D, Bueschel D, et al. *Borrelia burgdorferi* OspC protein required exclusively in a crucial early stage of mammalian infection. Infect Immun. 2006;74(6):3554-64.

8. Stewart PE, Thalken R, Bono JL, Rosa P. Isolation of a circular plasmid region sufficient for autonomous replication and transformation of infectious *Borrelia burgdorferi*. Mol Microbiol. 2001;39(3):714-21.
